# Mechanistic mathematical modeling of abscopal effect reveals mechanisms of off-target tumor response

**DOI:** 10.3389/fimmu.2026.1769229

**Published:** 2026-03-16

**Authors:** Andreas G. Hadjigeorgiou, Yiannis Roussakis, Constantinos Zamboglou, Triantafyllos Stylianopoulos

**Affiliations:** 1 Cancer Biophysics Laboratory, Department of Mechanical and Manufacturing Engineering, University of Cyprus, Nicosia, Cyprus; 2 Department of Medical Physics, German Oncology Center, Limassol, Cyprus; 3 Department of Radiation Oncology, German Oncology Center, European University Cyprus, Limassol, Cyprus; 4 Department of Radiation Oncology, University of Freiburg - Medical Center, Freiburg, Germany; 5 German Cancer Consortium (DKTK), Partner Site Freiburg, Freiburg, Germany

**Keywords:** abscopal effect, cancer-immune interaction, lymph node irradiation, mathematical modelling, radiotherapy and immunity

## Abstract

**Introduction:**

Local radiotherapy rarely triggers regression of distant, non-irradiated tumors (the “abscopal” effect), but this outcome is unpredictable because it depends on interacting processes, such as antigen release, antigen presentation, T-cell priming and trafficking, and lymphoid health. To study these interactions quantitatively and identify dominant mechanisms that control off-target tumor responses, we built an integrated physiologically based pharmacokinetic - quantitative systems pharmacology (PBPK-QSP) model.

**Methods:**

The PBPK-QSP model tracks immune (dendritic cells, M1/M2 macrophages, Tregs, naïve and effector CD8^+^ T cells, Antigen Presenting Cells) and tumor cell populations across body compartments that include a local (irradiated) and distant tumor, along with major organs, as well as the blood and lymph circulations. Radiotherapy is considered to have local effects on direct tumor cell killing, and indirectly by releasing tumor-associated antigens that induce immune cell priming, which drives the abscopal effect. Furthermore, we tested how lymph-node irradiation can affect immune cell priming. The model was calibrated against pertinent preclinical data, and sensitivity and correlation analyses were performed to investigate the mechanisms of off-target tumor response.

**Results:**

Four mechanisms dominate outcome variability: antigen capture/processing by phagocytes (i.e., dendritic cells and macrophages), clearance of dead-cell debris and antigens, and naïve T-cell regenerative capacity in lymph nodes. Phagocytic and clearance rates have context-dependent effects, too fast shortens the antigen-priming window, and too slow results in less overall antigen-priming. Lymph-node irradiation shifts the dependence of immune response to T-cell recovery, which becomes the dominant mechanism. The model also highlights that impaired tumor vascular permeability can constrain effector infiltration and mute intratumoral CD8^+^ T cells differences between the local and distant tumor despite systemic activation.

**Discussion:**

The PBPK-QSP model identifies specific, actionable mechanisms controlling abscopal responses and suggests three complementary strategies to increase the chance of abscopal responses: i) optimize radiotherapy dose/fractionation to maximize immunogenic antigen release while sparing lymphoid tissue when possible, ii) combine radiotherapy with interventions that prolong productive antigen presentation and modulate debris clearance, and iii) protect/restore lymphoid regenerative capacity.

## 1 Introduction

The abscopal effect describes the rare but clinically and biologically significant phenomenon in which local radiotherapy delivered to a single lesion leads to regression of metastatic lesions at distant, i.e., non-irradiated sites within the same host (1). The term was first introduced by R. H. Mole in 1953 to describe the phenomenon in which local irradiation can sometimes induce systemic effects beyond the treated field (1). Over the last two decades, with the increased applications of immunotherapy, a large body of preclinical and clinical work has implicated the immune system as the principal mediator of abscopal responses (2, 3). Local tumor irradiation can increase antigen availability, converting the irradiated lesion into an *in situ* vaccine that primes adaptive antitumor T-cells, which in turn traffic to and control distant metastatic lesions (3, 4). Importantly, combinations of radiotherapy with immune checkpoint inhibitors (ICIs) and other immunomodulatory agents have produced reproducible abscopal phenomena in experimental models and clinical case reports (5–9), showing that radiation and immunotherapy can synergistically convert sporadic abscopal occurrences into an exploitable therapeutic strategy.

The abscopal effect has been observed in various cancer types, including non-small cell lung cancer (NSCLC) (10, 11), renal cell carcinoma, hepatocellular carcinoma, lymphoma, and melanoma (12). The mechanism underlying this effect involves the local tumor ablation induced by radiotherapy, which causes the release of damage-associated molecular patterns and tumor-associated antigens (TAAs) (7, 13). These antigens are then presented to dendritic cells (DCs), leading to the development of a systemic antitumor immune response (7, 13). Despite these conceptual and translational advances, the abscopal effect remains unpredictable in the clinic where it is not always effective for all cancer patients due to the mechanistic complexity. Factors such as dose, fractionation, timing, tumor microenvironment (TME) properties, and systemic immune status all contribute to whether a local treatment yields a distant response or not. Preclinical studies have shown that these variables matter, for example, certain fractionation schemes synergize with ICI to elicit abscopal control, whereas single large doses do not (5). The interplay of the underlying mechanisms that affect the abscopal effect underscores the need for quantitative tools to explore parameter space and generate testable hypotheses.

Mathematical models of tumor–immune dynamics provide rigorously quantitative means to integrate heterogeneous biological data, test mechanistic hypotheses, and predict treatment outcomes under controlled *in silico* perturbations (14–19). Several mathematical models focus on the effect of the TME and treatment scheduling and have been developed to describe tumor growth and radiotherapy response across cancer types (20–29). Examples include models for head and neck (20, 25, 26, 29), non-small-cell lung (21), uterine cervical (22, 23), glioma (27, 28) and prostate (29) cancers. Model formulations range from subpopulation of oxygenated, hypoxic, and damaged cells (20, 26); logistic or Gompertzian tumor growth models coupled to radiation-induced tumor burden reduction (21, 23); and extensions of the linear–quadratic framework that incorporate radiotherapy memory (22), cell repopulation (22), or microenvironmental effects (24, 29); to approaches explicitly including cancer-stem cells (25), 3-D anisotropic diffusion tensors (27), or Monte Carlo/agent-based simulations with cell-cycle dynamics and p53 mutational status (28). Overall, the literature consistently highlights oxygenation status (20, 23, 25, 26, 29), radiosensitivity (20), and treatment fractionation strategy (25, 29) as key determinants of tumor control across modeling approaches.

Furthermore, previous mathematical approaches explicitly coupled radiotherapy with immune dynamics and reached conclusions for systemic immune response (abscopal effect) (30–33). One such model tracked tumor growth, radiation-induced antigen release, checkpoint inhibition, and primary and secondary (memory) immune responses, allowing exploration of how radiotherapy timing relative to immune checkpoint blockade alters the probability of generating a systemic (abscopal) response and the establishment of long-term immune control due to the secondary immune response (30). A Gompertz-based tumor growth model with explicit terms for radiotherapy, immunotherapy and their synergy, was fitted to experimental data with immunotherapy and radiotherapy (31). A small number of effective parameters reproduced experimental data, and at a specific immune activation state, the model predicted abscopal systemic regressions. Another model used four variables, including tumor volume, immunocompetent cell density, tumor antigen, and radiotherapy repair, to reproduce the three classical phases of cancer immunoediting (elimination, equilibrium, escape) after radiotherapy (32). Another study simulated tumor growth using the Gompertz law and included time-dependent effective terms that model both direct ultra-high-dose (FLASH) radiotherapy rate killing on the primary tumor and delayed systemic immune response on the metastases. They found that FLASH can produce a fast initial tumor killing and a delayed immune-mediated regression on the tumor metastases (33). Collectively, these studies used aggregated model variables to simulate antigen release and immune activation with radiotherapy timing and dose effects to simulate the synergy of radiotherapy with systemic immunity.

In this study, we extend our previously validated and published mathematical model that simulates tumor growth, immune interactions, and immune cells trafficking throughout the body (34) by considering two tumor compartments (local/irradiated and distant/non-irradiated) and the effects of radiotherapy applied to one of them. The current model is designed to (i) reproduce experimental data that show abscopal effects, (ii) identify parameters and mechanisms that maximize the probability of an abscopal outcome, and (iii) generate mechanistic predictions that can guide focused preclinical experiments. The designation “distant” reflects the structure of the calibration dataset (two spatially separated subcutaneous tumors with only one irradiated) (35) but in general the second compartment represents any non-irradiated tumor region that is immunologically coupled to the irradiated site through systemic blood and lymphatic trafficking. Thus, the non-irradiated compartment could equally represent a physically distant metastasis or an adjacent tumor region. By explicitly coupling two tumor compartments through a shared immune system, our framework offers a tractable, hypothesis-driven platform to quantify how local therapy can elicit systemic antitumor immunity and to explore strategies for reliably inducing clinically meaningful abscopal responses. Unlike earlier ordinary differential equation (ODE)-based or phenomenological models that aggregate immune populations and treat tumors as single well-mixed compartments, our framework explicitly couples a validated physiologically based pharmacokinetic (PBPK) anatomical scaffold to a mechanistic quantitative systems pharmacology (QSP) core (34, 36, 37). This enables resolution of immune-cell trafficking across twelve organ compartments and therefore captures physiological transport, extravasation and lymphatic recirculation of immune cells at organ-level resolution. Mechanistically the model tracks nine distinct cell populations, explicitly models phagocytosis-driven antigen presenting cells (APCs) formation from viable and dead tumor cells (tumor cell debris), and routes APCs via lymphatics to draining lymph nodes where naïve-to-effector T cell conversion is computed and allows direct simulation of how local antigen release after radiotherapy produces systemic priming. Methodologically we also implement radiotherapy as a linear quadratic (LQ) -based cytotoxic term that simultaneously drives antigen release and permits direct lymph-node irradiation (with radiation-induced depletion of APCs and T cells), which in turn makes it possible to study field design effects on abscopal outcomes. This modeling strategy is a contrast to prior models that either omitted whole-body transport, represented immune activation with a single phenomenological term, or ignored damage of draining lymph nodes (DNL).

## 2 Materials and methods

### 2.1 PBPK-QSP model of immune response, radiotherapy, and abscopal effect in solid tumors

**Figure 1:**
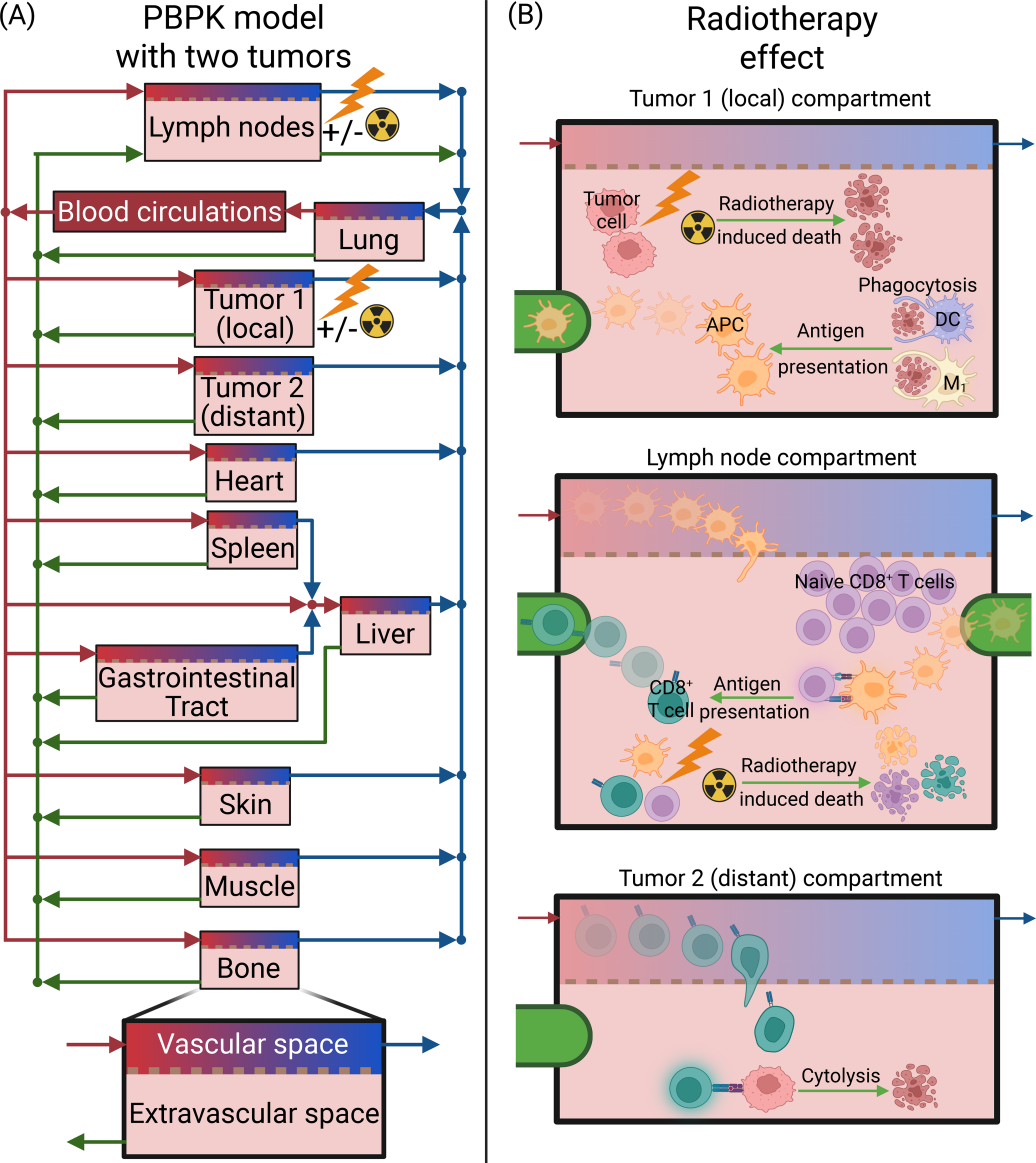
Schematic of the PBPK-QSP model used to simulate the tumor growth of two separate tumors (local and distant), immune response, and the effect of radiotherapy on the one/local tumor compartment and on the lymph nodes. **(A)** The PBPK model with the compartments of the body, which are connected by the blood circulation (blue and red arrows) and lymph circulation (green arrows), including two tumor compartments. **(B)** the effect of radiotherapy on the tumor compartment, which releases tumor-associated antigen. The debris and tumor-associated antigen from the dead cancer cells are phagocytosed by dendritic cells (DC) and macrophages type 1 (M1), which can become antigen-presenting cells (APC). APCs are transported to the lymph nodes through lymphatic vessels, where they activate the naïve CD8^+^ T cells against the tumor antigen. When radiotherapy is also applied to the lymph nodes induces death to APCs and naïve and effector CD8^+^ T cells. The effector CD8^+^ T cells enter the circulation through the lymphatic vessels and can induce cytolysis on cancer cells in both tumor compartments. Created in BioRender. Stylianopoulos, T. (2026) https://BioRender.com/5rcf258.

The current model is based on a validated framework that combines a PBPK model for anatomical resolution of immune cell trafficking with a QSP approach for mechanistic modeling of immune-tumor interactions (14, 34). It forms a comprehensive computational platform that captures both biodistribution and functional dynamics of immune populations across the organism. The model incorporates nine cell types: DCs, macrophages type 1 and 2 (M1 and M2), regulatory T cells (Tregs), naïve and effector CD8^+^ T cells (TN and TE1, respectively), APCs, and viable and dead tumor cells (Tv and Td). Among these, six cell types (DCs, M1, M2, Tregs, TE1, and APCs) circulate through the physiological compartments, while TN, Tv, and Td are confined to specific tissues, TN at lymph nodes and Tv and Td at the local and distant tumor compartments. The PBPK structure spans ten organ compartments: lungs, liver, gastrointestinal tract, spleen, heart, kidneys, skin, muscle, bone, and lymph nodes, similar to previous PBPK models (34, 36–39), plus two anatomically distinct tumors linked through both blood and lymphatic circulations, as depicted in **Figure 1A**. Each organ is divided into vascular and extravascular sub-compartments, enabling simulation of immune cell extravasation, interstitial residence, and lymphatic return. The vascular network governs rapid systemic transport through the bloodstream, whereas the lymphatic network mediates recirculation of immune cells to the lymph nodes.

Population conservation for each cell type is calculated through a system of coupled ODEs that track all relevant biological processes, including cellular influx and efflux between compartments, proliferation, death, cytolytic killing of tumor cells by effector T cells, phagocytosis of dead tumor debris and viable tumor cells by DCs and M1 macrophages, activation of immune cells against tumor antigens, suppression by regulatory cells such as Tregs and M2 macrophages, and transport between vascular and extravascular spaces. Each circulating population is represented by two equations per organ, corresponding to vascular and extravascular dynamics, ensuring population balance across the entire physiological network.

Within the tumor compartments, the extravascular regions explicitly describe the interactions between tumor and immune cells. Viable tumor cells proliferate and die through the cytotoxic activity of effector CD8^+^ T cells. The accumulation of dead tumor cells provides a dynamic source of debris and TAAs, which are engulfed by phagocytic DCs and M1 macrophages. These interactions generate antigen-presenting cells that drive the initiation of a systemic anti-tumor immune response. The evolving balance among proliferation, death, and immune-mediated cytolysis continuously updates tumor volume, thereby coupling the biophysical and immunological dynamics of the tumor microenvironment.

The model also incorporates the effects of radiotherapy to investigate both local and systemic immune responses associated with the abscopal effect. As shown in **Figure 1B**, radiotherapy applied to one tumor compartment induces direct cell death, transforming viable tumor cells into a pool of dead cells that release TAAs. These antigens are phagocytosed by DCs and M1 macrophages, which subsequently mature into APCs. The newly generated APCs migrate from the irradiated tumor through the lymphatic vessels to the draining lymph nodes, where they activate naïve CD8^+^ T cells against the tumor antigen, giving rise to effector T cells (TE1). These activated TE1 cells reenter systemic circulation via lymphatic drainage and home to both tumor compartments, where they mediate cytolytic killing of tumor cells. This process reproduces the central biological cascade underlying the abscopal effect, in which local radiotherapy triggers systemic anti-tumor immunity and distant tumor regression. The model also accounts for scenarios where the lymph nodes receive direct radiation, resulting in radiation-induced depletion of APCs, TN, and TE1 populations. This feature enables exploration of how radiotherapy can either potentiate or suppress systemic immune activation and consequently alter the probability of observing an abscopal response.

Numerical integration of the system is performed using an implicit Euler (backward differentiation) scheme implemented in COMSOL Multiphysics 5.6. The solver employs adaptive time stepping with a relative tolerance of 10^-6^. Parameterization of the model is based on previously published and validated models from PBPK and QSP studies of immune dynamics (34, 36, 37). The new parameters that were introduced to simulate the radiotherapy and abscopal effect were derived by model validation with previously published experimental data (35). The radiotherapy effect was modeled by employing the LQ model (20, 23, 25, 26, 29, 31). For the validation of the new dataset, all the parameters related to the tumor cells and their interactions with immune cells were kept the same across all treatment groups except two parameters. The activation rate constant of naïve CD8^+^ T cells ( $k_{TN}$) and the cytolytic potential of effector CD8^+^ T cells ( $k_{rc1}$) changed between radiotherapy-treated groups and control. These two key parameters ( $k_{TN}$ and $k_{rc1}$) were assumed to be affected by radiotherapy due to the increased tumor antigen that stimulate the adaptive immune system via sensing pathways (e.g., type I interferon/STING) inflammatory cytokines (e.g., IFN-γ, IL-12) that were not directly modeled and improved antigen recognition (7, 40–42).

The complete set of equations is described in the **Supplementary Material** where the core equations of the PBPK-QSP model are similar for all cell types while the different terms related to specific cell types (e.g. activation, suppression etc.) are summarized in **Supplementary Table 1**. A large number of parameters related to the PBPK-QSP model including transport of cells, flows, and volumes come from previous validated PBPK models (34, 36, 37) while only the parameters related to the current cancer cell type, cancer-immune interactions and radiotherapy are estimated based on pertinent experimental data. These new parameters and their values with the highest probability are presented in **Supplementary Table 2** and the parameters adopted by previous publications (34, 36, 37) in **Supplementary Table 3**.

### 2.2 Model calibration and estimation of unknown parameters

A subset of mechanistic parameters that directly determine tumor intrinsic growth, tumor–immune interactions, phagocytic handling of antigen/debris, lymphoid regeneration, and radiosensitivity were calibrated. The fitted parameters are summarized in **Supplementary Table 2** and consist of: intrinsic tumor growth rate $lg_{tumor}$, initial tumor volume $V_{i_{tumor0}}$, dead-cell clearance rate $Clear_{c_{dead}}$, phagocytic rate constants for viable cancer cells by DCs and APCs $A_{c_{DC}}$, and by M1 macrophages $A_{c_{M1}}$, phagocytic rate constants for dead cancer cells (debris) by DCs and APCs $A_{c_{{\text{dead}}_{DC}}}$, and by M1 $A_{c_{{\text{dead}}_{M1}}}$, naïve CD8^+^ T cells proliferation $k_{{\text{prol}}_{TN}}$, linear–quadratic radiosensitivity parameters for cancer cells $a_{c}$ (α) and $b_{c}$ (β), linear–quadratic radiosensitivity parameters for immune cells in lymph nodes ( $a_{IM}$, $b_{IM}$), naïve to effector CD8^+^ T cell activation rate $k_{TN}$, and the effector CD8^+^ T cell cytolytic rate of cancer cells $k_{rc1}$. All other PBPK transport and physiological parameters were held fixed and taken from previously validated sources (**Supplementary Table 3**) (34, 36, 37). It must be noted that the fitted parameter values were constrained to be identical across treatment arms except for $k_{TN}$ and $k_{rc1}$. $k_{TN}$ and $k_{rc1}$ were allowed to differ between control and radiotherapy-treated groups (RT and RT+LN-RT) to capture the main downstream immunological consequences of radiotherapy.

This assumption is relevant because radiotherapy increases tumor antigen availability and activates innate sensing pathways (e.g., type I interferon/STING) that enhance dendritic-cell maturation and CD8^+^ T cell priming, it also augments effector CD8^+^ T cell functional quality via inflammatory cytokines (e.g., IFN-γ, IL-12) and improved antigen recognition (7, 40–42). Because our mechanistic model does not explicitly represent the full cytokine network, we capture the net treatment-dependent effects by allowing the naïve-to-effector activation rate $k_{TN}$ and the effector cytolytic rate $k_{rc1}$ to vary between controls and radiotherapy-treated groups. All other parameters governing trafficking, phagocytosis and baseline tumor kinetics were kept identical across groups to isolate RT-specific immune modulation.

For calibration, we used individual mouse tumor-volume time curves extracted from the published preclinical study (35). For each subject, tumor volumes were digitized and organized by treatment group and tumor (T1, T2). The fitting objective minimized the sum-of-squared errors (SSE) between model predictions and two observables derived from the experimental data: (i) tumor-volume time series and (ii) an empirical tumor growth rate approximated by a central difference of the tumor-volume measurements. The same radiation dose, fractionation and timing used in the experiments were implemented in the simulations (35).

Parameter estimation was performed by minimizing the objective function that compares model-predicted and experimentally measured tumor observables. To ensure positivity of fitted parameters, optimization was carried out in log-space, namely the optimization algorithm optimizes the vector $\text{ln}\left(\theta_{i}\right)$, where $\theta_{i}$ are the fitted parameters, and are used in the model to run the simulations and calculate the simulated data. Bootstrap resampling was used to quantify parameter uncertainty. For each bootstrap replicate we sampled tumor growth curves with replacement within each treatment group and tumor number (T1 and T2) so that group sizes and tumor time-series were preserved, assembled the resampled dataset, and refit the model with the same optimization algorithm.

The optimization algorithm used MATLAB’s genetic algorithm (ga, Global Optimization Toolbox) with a population-based, multi-individual search. The initial population matrix for each GA run included the baseline solutions from previous fits, the baseline optimum and prior bootstrap optima are retained and passed as initial population to accelerate convergence across replicates. To avoid hard truncation of the search space, lower and upper bounds for the ga were set adaptively from the current initial population: the per-parameter lower bound was the minimum value in the initial population minus up to 10% in log space (randomized per run) and the upper bound was the maximum value in the initial population plus up to 10% in log space (randomized per run). This stochastic ±10% expansion reduces the chance that the search is trapped at preset bounds. In total 50 bootstrap refits were performed, and optimal parameters were recorded and used to estimate the average, median, standard deviation, 90% confidence intervals (CI) of the simulated curves. A pseudo code that describes the procedure in more detail is provided in the **Supplementary Material**.

## 3 Results

### 3.1 Model calibration with experimental data

**Figure 2:**
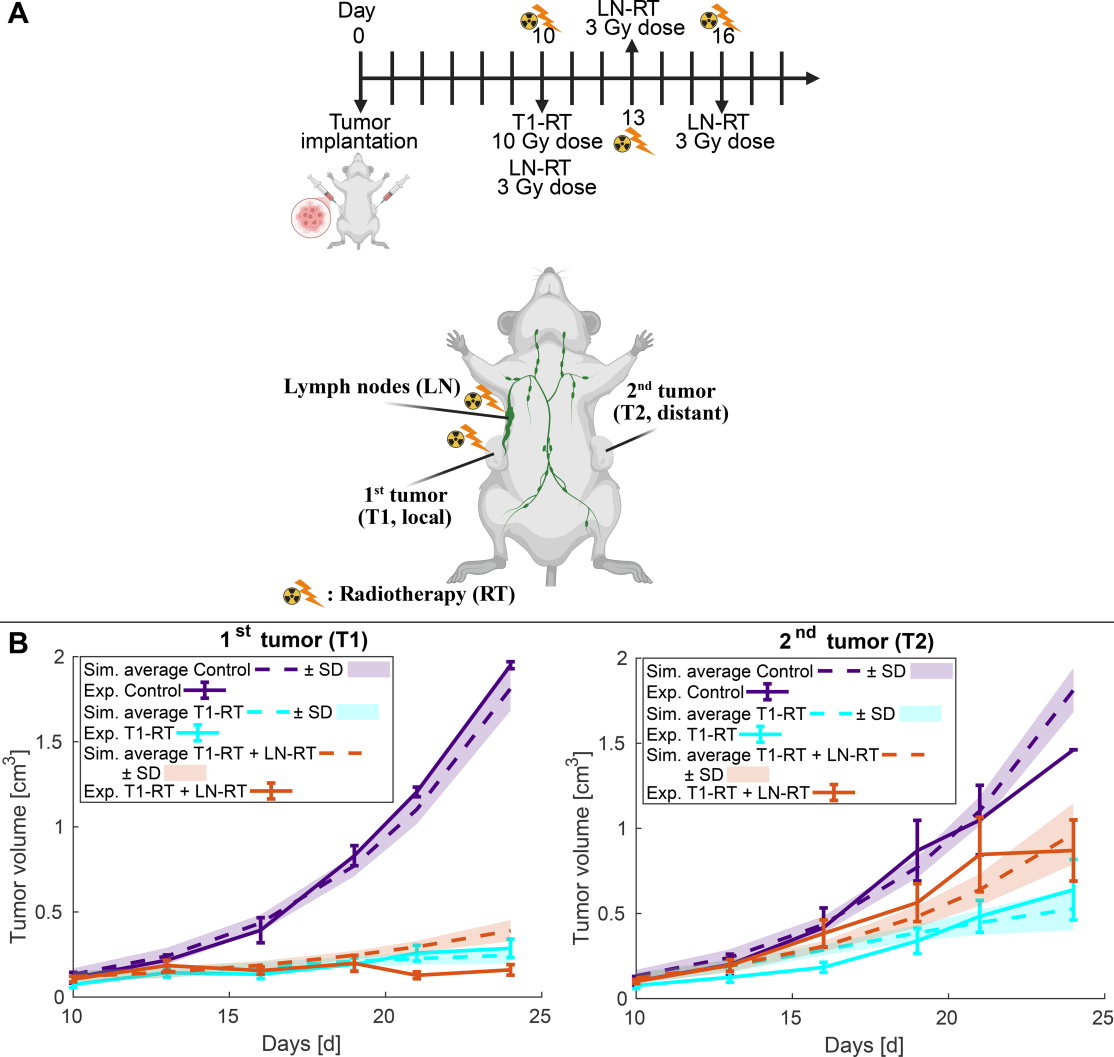
Comparison of model predictions with experimental tumor growth curves in bilateral subcutaneous flank B16F10GP melanoma tumors. **(A)** The schematic illustrates the experimental and simulated protocol, which are identical to the study (35). Mice bearing two independent bilateral melanoma tumors (T1 and T2) were subjected to three treatment groups according to the experimental protocol: control (no treatment), localized radiotherapy applied to the first tumor only (T1-RT), or combined radiotherapy applied to both the first tumor and the tumor-draining lymph nodes (T1-RT + LN-RT), according to the doses and schedule presented in the timeline. **(B)** Tumor growth curves for the 1^st^ tumor, which might be irradiated, and the 2^nd^ non-irradiated tumor. The average experimental tumor growth data (Exp.) calculated from individual tumor growths (35) are shown as continuous lines (35) (± standard error) n=8, while the corresponding average of model simulations (Sim.) from the optimal sets of parameters derived from bootstrap resampling and refit process are represented by dashed lines (± standard deviation). Illustration of panel **(A)** is Created in BioRender. Stylianopoulos, T. (2026) https://BioRender.com/5rcf258.

To evaluate and calibrate the new parameters (**Supplementary Table 2**) of the proposed model, we compared its predictions with published *in vivo* data obtained from mice bearing bilateral subcutaneous flank B16F10GP melanoma tumors (35). In this experimental system, animals developed two anatomically different tumors, referred to as T1 and T2, where only T1 might receive radiotherapy (local tumor). This setup allows the study of both local and distant anti-tumor effects following localized irradiation (**Figure 2A**). Three treatment conditions were investigated: (i) untreated control animals (Control), (ii) localized radiotherapy applied exclusively to the first tumor (T1-RT), and (iii) combined radiotherapy delivered to the first tumor and to the corresponding tumor-draining lymph nodes (T1-RT + LN-RT). The experimental and the simulated protocol are identical and are presented in **Figure 2A**, T1 received 10Gy radiotherapy on day 10 (groups T1-RT and T1-RT + LN-RT) and tumor draining lymph nodes received 3Gy radiotherapy on days 10, 13 and 16 (group T1-RT + LN-RT). Tumor growth data from both tumors were used to validate the model and are presented along with the simulated data in **Figure 2B**.

Although the experimental tumor growth curves have high variability (35), the model successfully reproduced the trend between different groups, as shown in **Figure 2B** where the average simulated curves (± standard deviation) from the optimal sets of parameters (see their distribution in **Supplementary Figure S1**) derived from bootstrap resampling and refit process are presented. In the control group, both T1 and T2 exhibited exponential growth consistent with the absence of therapy-induced cell death or immune activation. When radiotherapy was administered to T1 alone, the model predicted a marked reduction in the volume of the irradiated tumor due to radiation-induced tumor cell death, consistent with experimental observations. In contrast, the contralateral tumor (T2) displayed a slower growth rate than the control, reflecting a measurable systemic immune response triggered by antigen release from the irradiated site.

When radiotherapy was extended to include both the primary tumor and the tumor-draining lymph nodes (T1-RT + LN-RT), the model predicted a distinct alteration in immune dynamics. Although the direct irradiation of lymph nodes does not significantly affect the growth of T1 (35), but induces collateral damage to APCs and CD8^+^ T cells within the lymphoid tissue. This resulted in a partial suppression of systemic immunity, leading to a less pronounced abscopal response in the distant tumor (T2) compared with the T1-RT-only group. The simulated growth curves accurately captured the experiments by reproducing both the rapid regression of the irradiated tumor and the modest regression of the distant tumor, and how irradiated tumor-draining lymph nodes affect tumor response (**Figure 2A**). The accuracy of model validation is also shown in **Supplementary Figure S2** where all the simulated individual curves and the median and 90% of CI from the 50 bootstrap sampling and model refit are presented.

Overall, the model demonstrated strong quantitative agreement with experimental tumor growth data, reproducing the distinct temporal patterns of local control and systemic immune modulation across treatment groups. This validation confirms that the combined PBPK-QSP framework can mechanistically represent key biological processes underlying radiation-induced immune activation and the emergence or suppression of the abscopal effect. These results support the model’s predictive capacity which were achieved by keeping all parameters, related to tumor growth, immune-tumor interactions and radiotherapy, consistent across treatment groups and varying only the two key parameters ( $k_{TN}$ and $k_{rc1}$) with radiotherapy (see **Supplementary Tables 2**, **3**). The latter two parameters were assumed to be affected due to the increased tumor antigens that stimulate the adaptive immune system. Subsequently, the model was used for exploring what parameters and conditions can induce or suppress the abscopal effect.

### 3.2 Local sensitivity of model parameters and their effect on tumor growth

To evaluate the effect of the parameters that were used for calibration and identify key determinants of treatment response, a local sensitivity analysis was performed by varying one parameter at a time. All optimal parameters from the bootstrap sampling and refit process (see Materials and Methods section) were varied over two orders of magnitude (0.1×–10× the baseline value). The optimal parameters were multiplied by 10, 1.5, 1.1, 0.9, 0.5, and 0.1 to test their effect close to the optimal value and far from it, and if their effect was preserved in this range. In the current section, results focused on four biologically significant parameters: i) the phagocytic potential of DCs and APCs ( $A_{c_{DC}}$), which determines the efficiency of antigen capture and presentation; ii) the cytolytic rate constant of CD8^+^ effector T cells ( $k_{rc1}$), which defines their tumor-cell killing capacity; iii) the activation rate of naïve CD8^+^ T cells by APCs ( $k_{TN}$), which controls the generation of effector cells in the lymph nodes; and iv) the proliferation rate of naïve CD8^+^ T cells ( $k_{prol_{TN}}$) in lymph nodes, which influences the overall expansion and regenerative potential of the lymphocytes.

**Figure 3:**
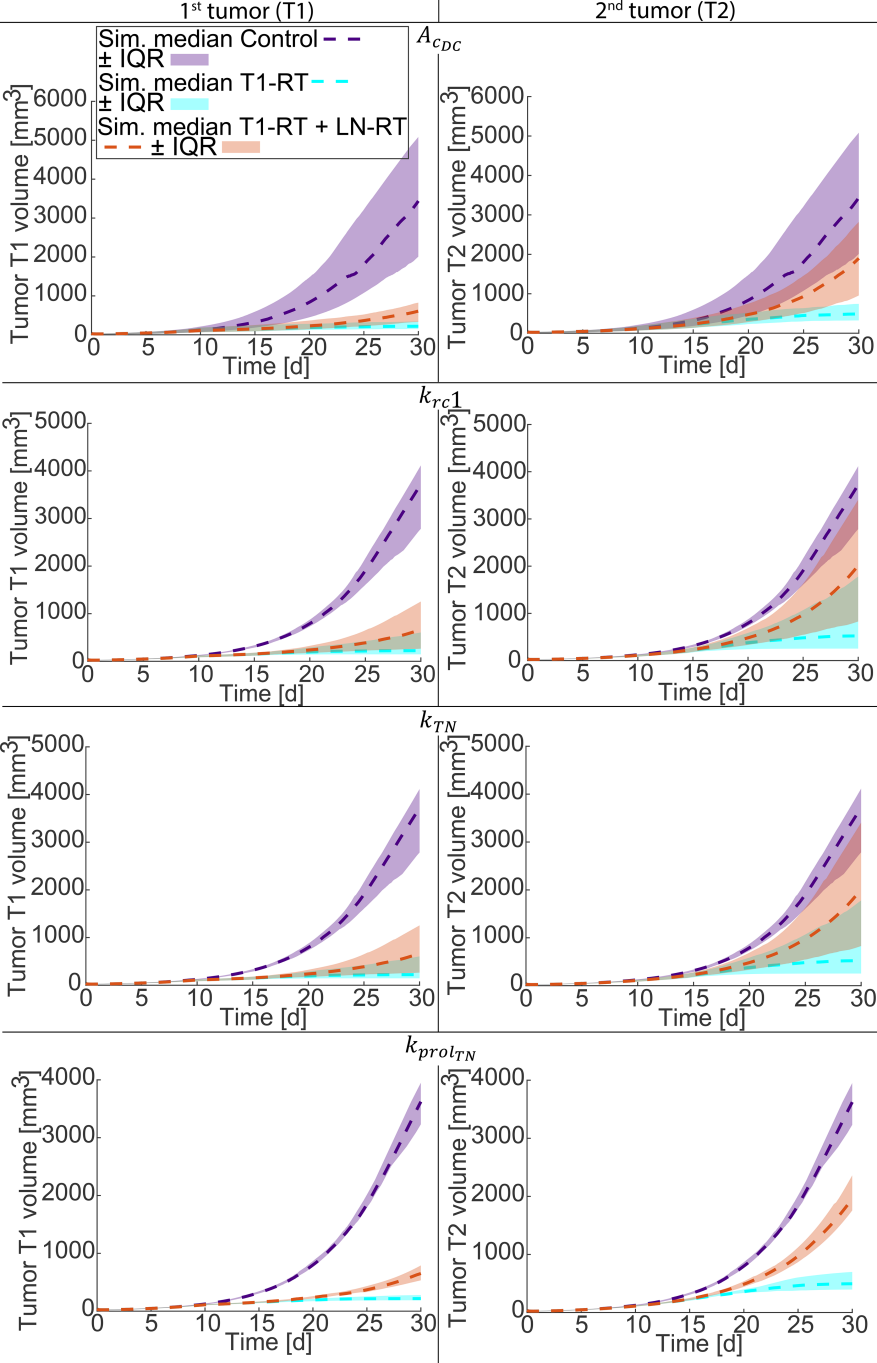
Sensitivity analysis of model predictions for tumor growth under different treatment conditions. Simulated tumor growth curves are shown for the Control, T1-RT, and T1-RT + LN-RT groups with the same protocol as in **Figure 2**. The shaded areas indicate the Interquartile Range (IQR) and dashed lines indicate their median curves, arising from variation of optimal parameters. Each parameter varied at 10, 1.5, 1.1, 0.9, 0.5, and 0.1 times its baseline value while all others were held constant at each optimal set. The parameters examined include the phagocytic potential of dendritic cells and antigen-presenting cells ( $A_{c_{DC}}$), the cytolytic capacity of CD8^+^ effector T cells ( $k_{rc1}$), the activation rate constant of naïve CD8^+^ T cells ( $k_{TN}$), and the proliferation rate constant of naïve CD8^+^ T cells ( $k_{prol_{TN}}$). The resulting variation illustrates how immune functional parameters modulate model variable in both irradiated (T1) and non-irradiated (T2) tumors.

The same treatment protocol and treatment groups (see **Figure 2**) were used to quantify the influence of parameters on the predicted tumor growth in both irradiated (T1) and non-irradiated (T2) tumors under each treatment condition. **Figure 3** presents the median simulated tumor growth curves (dashed lines) from all optimal parameters that were varied with their Interquartile Range (IQR, shaded areas) for the Control, T1-RT, and T1-RT + LN-RT groups. Median and IQR were used for presentation due to the high variation of the model predictions which results from the multiple optimal parameters and their variation over two orders of magnitude.

The model predicted that variations in $A_{c_{DC}}$, $k_{rc1}$, and $k_{TN}$ significantly impacted tumor dynamics across all three treatment groups, reflecting their central role in mediating systemic anti-tumor immunity. Increasing the phagocytic potential ( $A_{c_{DC}}$) enhanced the formation of antigen-presenting cells, thereby improving CD8^+^ T-cell priming and reducing tumor burden in both T1 and T2. Similarly, higher $k_{rc1}$ values intensified the cytolytic clearance of tumor cells, resulting in faster regression of the irradiated tumor and a stronger abscopal response. Enhanced naïve T-cell activation ( $k_{TN}$) also led to a consistent improvement in tumor control, as more effector T cells entered circulation and targeted both tumor sites. In contrast, changes in the naïve T-cell proliferation rate ( $k_{prol_{TN}}$) has lower effect than the other parameters showing an increasing effect on T2 in the T1-RT and T1-RT + LN-RT groups, potentially due to the increasing antigen presentation after radiotherapy. For T1-RT + LN-RT group radiotherapy applied to the lymph nodes can deplete local T-cell precursors and APCs, and the regenerative capacity of the naïve T-cell pool becomes a critical determinant of immune recovery and systemic efficacy.

Notably, the sensitivity analysis revealed that perturbations of all immune parameters produced larger variations in the growth trajectories of the non-irradiated tumor (T2) compared with the irradiated one (T1). This finding reflects the nonlinear and amplifying nature of systemic immune responses: the distant, untreated tumor depends entirely on immune activation initiated at the irradiated site, making it more susceptible to fluctuations in parameters governing antigen processing, T-cell activation, and proliferation. In contrast, T1 tumor dynamics are dominated by direct radiation cytotoxicity and are not influenced as much as T2 by immune parameters.

Overall, the analysis underscores the mechanistic interplay between radiation-induced local effects and immune-mediated systemic responses. Parameters associated with antigen presentation and T-cell function not only govern the extent of local tumor control but also critically determine the magnitude of the abscopal effect.

### 3.3 Local sensitivity of model parameters and their effect on CD8^+^ T cells

**Figure 4:**
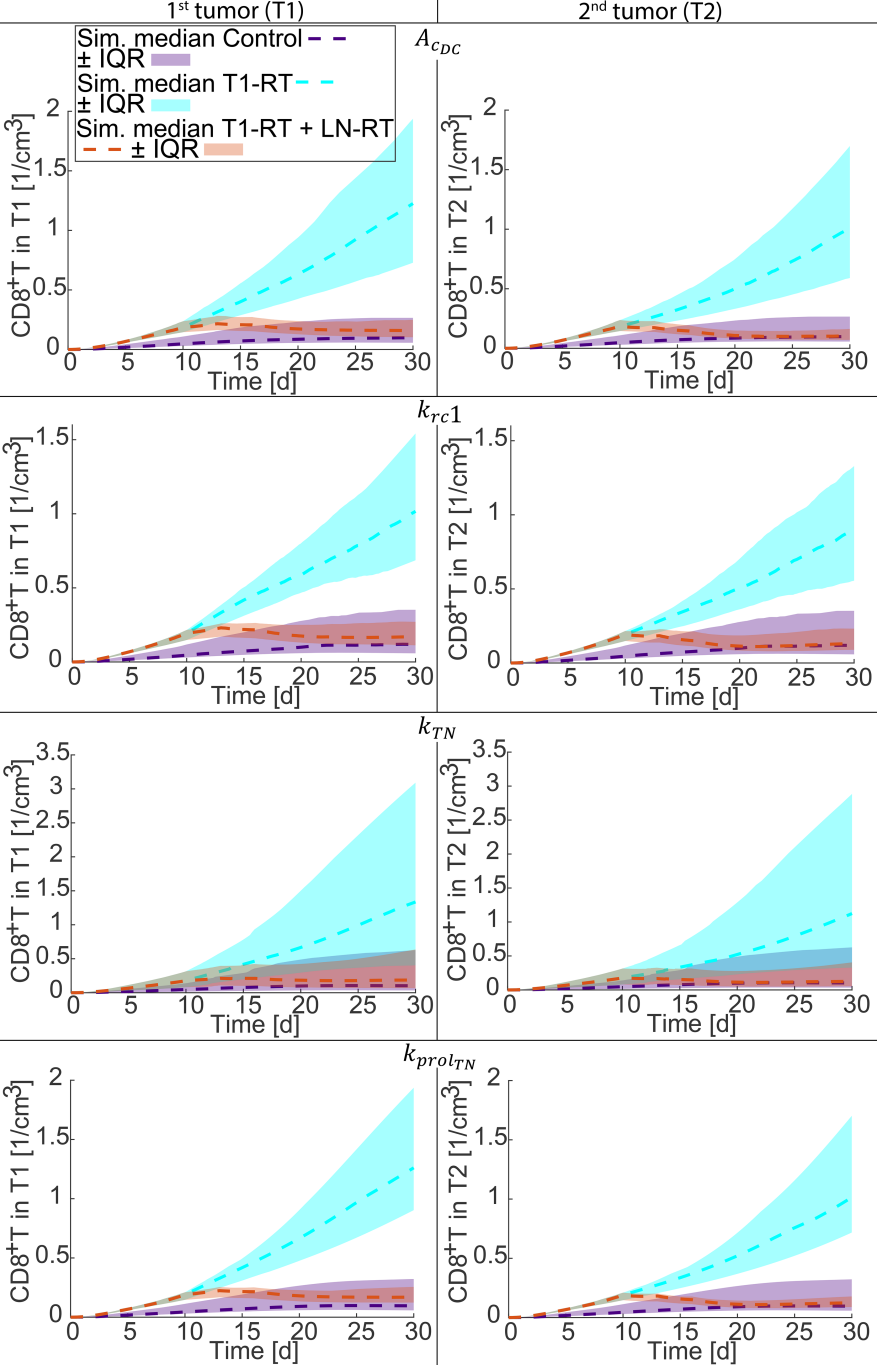
Sensitivity analysis of model predictions for CD8^+^ T cells under different treatment conditions. Simulated CD8^+^ T cells in T1 and T2 are shown for the Control, T1-RT, and T1-RT + LN-RT groups with the same protocol as in **Figure 2**. The shaded areas indicate the Interquartile Range (IQR) and dashed lines indicate their median curves, arising from variation of optimal parameters. Each parameter was varied at 10, 1.5, 1.1, 0.9, 0.5, and 0.1 times its baseline value while all others were held constant at each optimal set. The parameters examined include the phagocytic potential of dendritic cells and antigen-presenting cells ( $A_{c_{DC}}$), the cytolytic capacity of CD8^+^ effector T cells ( $k_{rc1}$), the activation rate constant of naïve CD8^+^ T cells ( $k_{TN}$), and the proliferation rate constant of naïve CD8^+^ T cells ( $k_{prol_{TN}}$). The resulting variation illustrates how immune functional parameters modulate tumor control in both irradiated (T1) and non-irradiated (T2) tumors.

Results of the sensitivity analysis for CD8^+^ T cells are presented in **Figure 4**, which provide additional insights into how immune-related parameters shape the systemic anti-tumor response under different radiotherapy conditions. The simulated temporal profiles of CD8^+^ T cells in both the irradiated (T1) and non-irradiated (T2) tumors were examined for the Control, T1-RT, and T1-RT + LN-RT treatment groups with the same protocol as previous (see **Figure 2A**) and the same parameters, $A_{c_{DC}}$, $k_{rc1}$, $k_{TN}$, and $k_{prol_{TN}}$. The shaded area represents the IQR, while dashed lines indicate the median CD8^+^ T cell response across simulations due to variation of each parameter across all optimal set from bootstrap resampling and refit process.

The results show that variations in $A_{c_{DC}}$, $k_{rc1}$, $k_{TN}$ and $k_{prol_{TN}}$ influenced CD8^+^ T cell populations across all treatment groups, indicating that these parameters play a universal role in shaping both local and systemic immune responses. Increases in the phagocytic potential ( $A_{c_{DC}}$) enhanced antigen uptake and presentation, resulting in higher levels of CD8^+^ T cell activation in both tumors. Similarly, higher $k_{rc1}$ values amplified the cytolytic feedback between effector cells and tumor antigens, indirectly sustaining T-cell expansion through antigen-driven activation. Modulating the activation constant ( $k_{TN}$) strongly affected the amplitude of CD8^+^ T cell accumulation, confirming that efficient activation in lymph nodes is a key determinant of the systemic immune response following radiotherapy. The proliferation rate constant of naïve CD8^+^ T cells ( $k_{prol_{TN}}$) affects CD8^+^ T cell populations by replenishing naïve CD8^+^ T cells in the lymph nodes. Between all groups only the T1-RT group shows an increasing accumulation of CD8^+^ T cells, while the Control group reaches a plateau and the T1-RT + LN-RT group struggles to recover after lymph node irradiation.

Despite these variations, the simulated CD8^+^ T cell concentrations within the irradiated (T1) and non-irradiated (T2) tumors were not markedly different. This modest inter-tumoral difference likely reflects transport limitations imposed by the tumor microenvironment. Specifically, the model incorporates cell extravasation parameters derived from previous work characterizing immune-cell migration through the abnormal vasculature of solid tumors (34). Such parameters capture the restricted permeability and structural heterogeneity typical of tumor blood vessels, which can constrain immune-cell infiltration even when systemic activation is robust. Consequently, while radiation and immune parameters modulate T-cell activation and systemic availability, impaired trafficking through abnormal vasculature can diminish local accumulation, leading to comparable CD8^+^ T cell densities in both tumor sites.

### 3.4 Quantification of total variance due to variation of model parameters

To quantify the effect of individual model parameters on tumor and immune dynamics, we computed a time-integrated total variance for each parameter using the one-at-a-time simulation ensembles generated from each bootstrap-optimal parameter set. For bootstrap replicate b and for a given parameter (swept through its test values as previously descripted, 10, 1.5, 1.1, 0.9, 0.5, and 0.1 times) we compute


$$TotVar_{j}^{\left(b\right)}=\sum_{i}1/T\int_{0}^{T}{\left(V_{i}^{\left(b\right)}\left(t\right)-{\bar{V}}^{\left(b\right)}\left(t\right)\right)}^{2}dt$$

where T is the time of integration, $V_{i}^{\left(b\right)}\left(t\right)$ is the model output (tumor volume or CD8^+^ T cell level) for the i-th value of the j-th parameter using the bootstrap baseline b, and ${\bar{V}}^{\left(b\right)}\left(t\right)$ is the time-dependent mean across those i simulations for replicate b. So, for each bootstrap-optimum we vary each parameter alone, compute the temporal variance of the resulting ensemble, and integrate/average it over the observation window to obtain ${\text{TotVar}}_{j}^{\left(b\right)}$. This design isolates the variation induced by parameter perturbation from the baseline differences across bootstrap optima.

**Figure 5:**
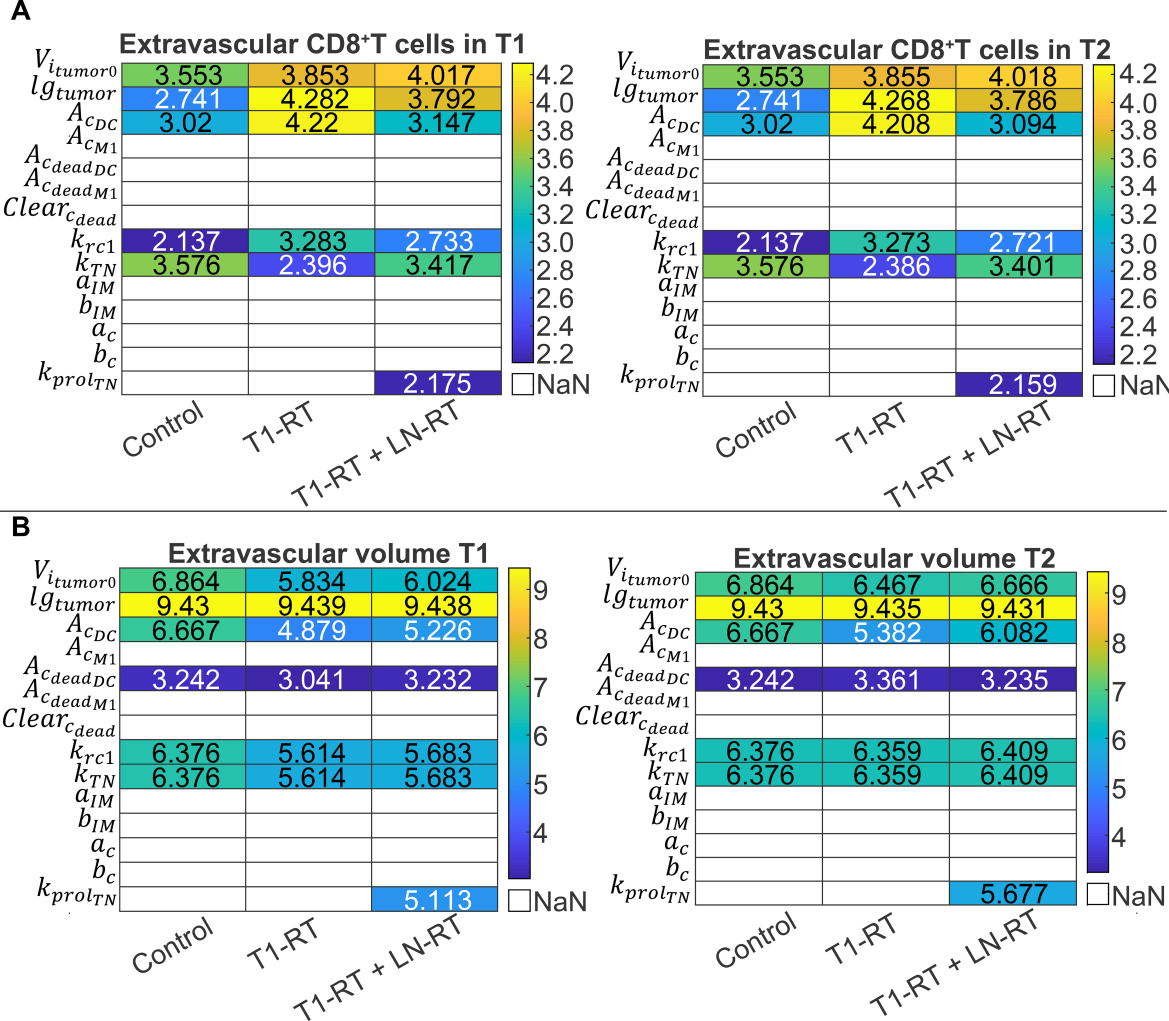
Quantification of parameter-induced total variance in model outputs. For each bootstrap-fitted baseline, one-at-a-time parameter variations were performed and a time-integrated total variance ( $TotVar_{j}^{\left(b\right)}$) was computed for **(A)** CD8^+^ T cells levels and **(B)** tumor volume for both the first (T1) and second (T2) tumor. Total variance values exceeding a predefined threshold ( $TotVar_{j}^{\left(b\right)}$ > 10) were considered significant and retained. For each parameter, the average significant total variance ( $\bar{TotVar_{j}}$) was then calculated across all bootstrap replicates to isolate variability driven by parameter perturbation rather than by differences among bootstrap-fitted optima. Heatmaps present the logarithm of the average significant total variance, illustrating the order of magnitude of parameter-induced variability. Parameters that never exceeded the threshold are shown as NaN.

To focus on robust, non-negligible effects we applied a significance threshold: within each bootstrap replicate, we retained only those $TotVar_{j}^{\left(b\right)}$ values that exceeded the threshold ( $TotVar_{j}^{\left(b\right)}>10$ which satisfies a change of at least one order of magnitude). For each parameter j we then averaged the retained (significant) ${\text{TotVar}}_{j}^{\left(b\right)}$ values over all bootstrap replicates to obtain a single quantity $\bar{TotVar_{j}}$ that summarizes the variation independent of the baseline. The base-10 logarithm of $\bar{TotVar_{j}}$ is displayed in the heatmaps (**Figure 5**) to compress dynamic range and facilitate visual comparison across parameters and outputs. Values that never exceeded the significance threshold across all bootstraps are plotted as NaN in the heatmap.

For CD8^+^ T cells (**Figure 5A**) in the local tumor (T1) without treatment (Control), the highest total variances were observed for the activation rate of naïve CD8^+^ T cells ( $k_{TN}$) and the initial tumor volume ( $V_{i_{tumor0}}$), followed by the phagocytic potential of DCs and APCs ( $A_{c_{DC}}$), tumor growth rate ( $lg_{tumor}$), and cytolytic rate constant of effector T cells ( $k_{rc1}$). When radiotherapy was introduced in T1 (T1-RT group), the rank order shifted slightly, with $lg_{tumor}$ coming first and $A_{c_{DC}}$ becoming more influential, followed by $V_{i_{tumor0}}$, $k_{TN}$, and $k_{rc1}$. In the combined T1-RT + LN-RT treatment, $\;V_{i_{tumor0}}$ dominated, followed by $\;lg_{tumor}$, $A_{c_{DC}}$, $k_{TN}$, $k_{rc1}$and $k_{prol_{TN}}$. The corresponding variances for CD8^+^ T cells in the distant, non-irradiated tumor (T2) were nearly identical to local tumor (T1), which likely reflects transport limitations of immune cells imposed by the tumor microenvironment.

These results indicate that parameters governing immune activation: $k_{TN}$ and antigen handling $A_{c_{DC}}$ consistently exert strong control over CD8^+^ T cell dynamics in all treatment conditions, while the initial tumor volume and growth rate modulate the antigenic load and therefore indirectly influence immune expansion. Interestingly, the parameter $k_{prol_{TN}}$ have significant effect only in the T1-RT + LN-RT group, which indicates that the naïve T-cell proliferation rate in lymph nodes become crucial when lymph nodes are irradiated.

Analysis of tumor volume variance (**Figure 5B**) revealed a stronger dependence on tumor-intrinsic parameters, particularly the tumor growth rate $lg_{tumor}$ and the initial tumor size $V_{i_{tumor0}}$. For the Control group, $lg_{tumor}$ dominated, followed by $V_{i_{tumor0}}$, $A_{c_{DC}}$, $k_{TN}$, $k_{rc1}$, and the phagocytic potential of DC and APCs for dead cancer cells $A_{c_{dead_{DC}}}$. When radiotherapy was introduced in T1 (T1-RT group), the hierarchy remained similar for the tumor-intrinsic parameters, with $lg_{tumor}$ as the most influential, followed by $V_{i_{tumor0}}$, while activation and killing potential of CD8^+^ T cells ( $k_{TN}$, and $k_{rc1}$) become more influential, than the phagocytic potential of DC and APC ( $A_{c_{DC}}$) for viable cancer cells, and $A_{c_{dead_{DC}}}$ remains the least influential parameter. In the combined T1-RT + LN-RT group the order of the influence of the parameters is the same as with the T1-RT group but the effect of phagocytic potential on DC and APC ( $A_{c_{DC}}$ and $A_{c_{dead_{DC}}}$) increased compared to the T1-RT group and $k_{prol_{TN}}$ appears to have an influence similar to $A_{c_{DC}}$, reflecting the stronger dependency of tumor control on immune response recovery when lymph nodes are irradiated. Similar patterns were observed for the second tumor (T2), with $lg_{tumor}$ consistently yielding the highest variance and immune-related parameters $k_{TN}$, $k_{rc1}$, $A_{c_{DC}}$, and $k_{prol_{TN}}$ showing higher influence in the T1-RT and T1-RT + LN-RT group compared to T1.

Collectively, these findings demonstrate that tumor volume variance is driven primarily by intrinsic tumor growth kinetics and baseline tumor burden, whereas CD8^+^ T cell variance is more sensitive to immunological parameters controlling activation and proliferation of T cells. Importantly, when T1 is irradiated, T2 shows higher dependency on parameters related to CD8^+^ T cell ( $k_{TN}$ and $k_{rc1}$) and when lymph nodes are also irradiated, the dependency on naïve T cells proliferation ( $k_{prol_{TN}}$) and phagocytic potential of DC and APCs for viable cancer cells ( $A_{c_{DC}}$) increased, indicating that the T cells and APCs recovery becomes a crucial factor for immune response, especially for non-irradiated tumors (T2).

Overall, the variance analysis reinforces the interplay between tumor-intrinsic and immune-mediated processes in determining treatment outcomes. Tumor growth rate and initial size primarily dictate baseline progression, while immune activation and proliferation parameters govern the amplitude and persistence of the antitumor immune response, which affects the non-irradiated tumors more than the irradiated.

### 3.5 Correlation of model parameters and differences between treatment groups

**Figure 6:**
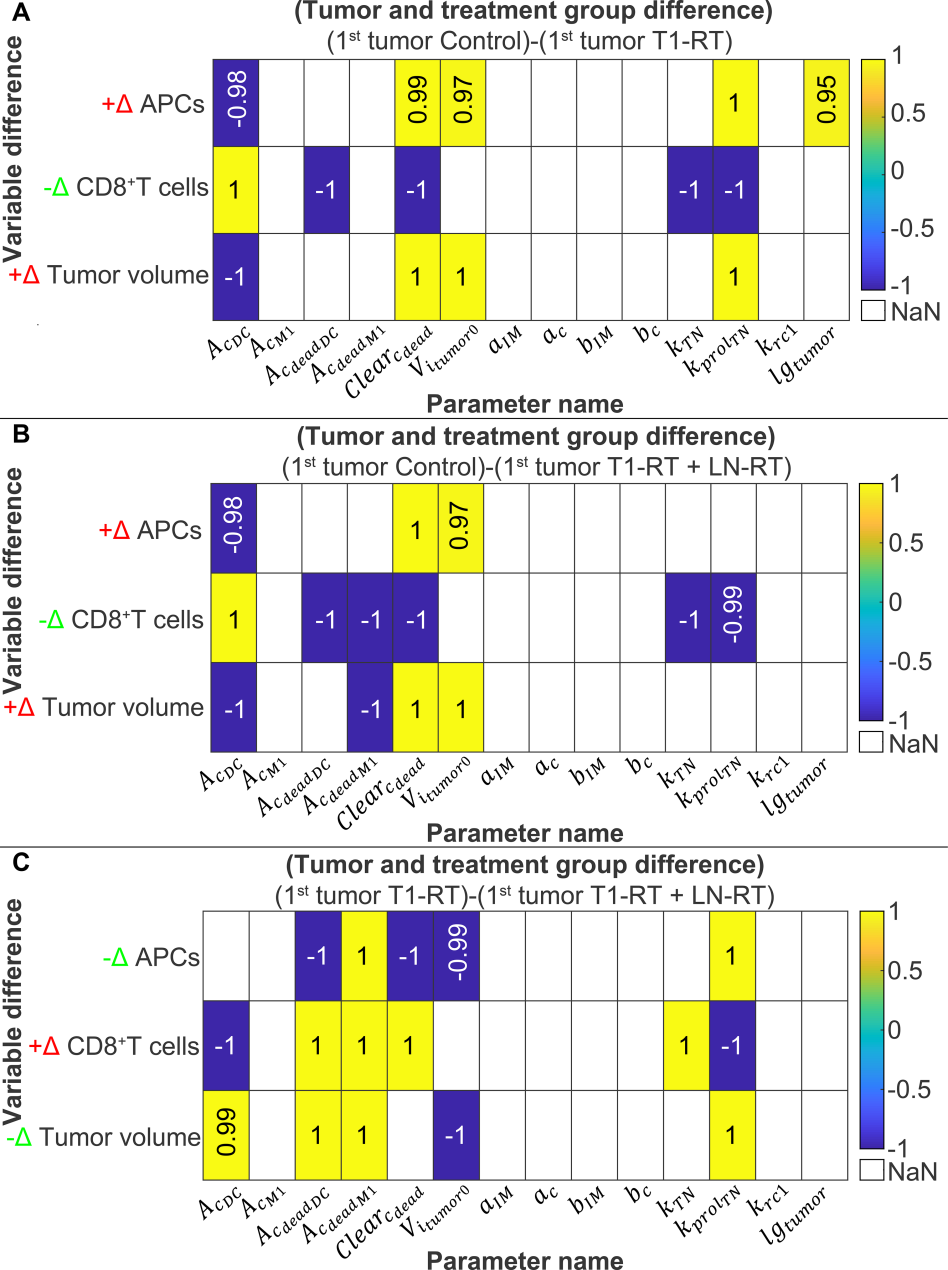
Correlation analysis between model parameter and inter-treatment differences in key immune and tumor variables within the 1^st^ local tumor (T1). Spearman’s rank correlation coefficients (ρ) were computed between one-at-a-time parameter perturbations and time-averaged differences in predicted antigen-presenting cells (APCs), CD8^+^ T cells, and tumor volume among treatment groups. Panels show correlations for differences **(A)** Control vs T1-RT, **(B)** Control vs T1-RT + LN-RT, and **(C)** T1-RT vs T1-RT + LN-RT. The sign indicator (+Δ or −Δ) denotes the direction of the mean difference between groups (e.g., +Δ indicates higher values in the first group listed). E.g., for the Control and T1-RT groups, a positive sign (+Δ) means the variable is higher in the Control than the T1-RT group, and a negative sign (-Δ) vice versa. Rows correspond to model variables and columns to model parameters. Only statistically significant correlations (p< 0.05) that were consistent across bootstrap refits are displayed, i.e. only those averaged ρ values that had the same sign across bootstraps and were statistically significant (p< 0.05) in the majority of bootstrap replicates (threshold: ≥70% of bootstrap refits) were presented. For variables with +Δ difference, positive correlations (yellow) indicate that increasing the parameter enhances the difference between treatment groups, whereas negative correlations (blue) indicate that increasing the parameter reduces the difference. For variables with -Δ difference, positive correlations (yellow) indicate that increasing the parameter reduces the difference between treatment groups, whereas negative correlations (blue) indicate that increasing the parameter enhances the difference.

**Figure 7:**
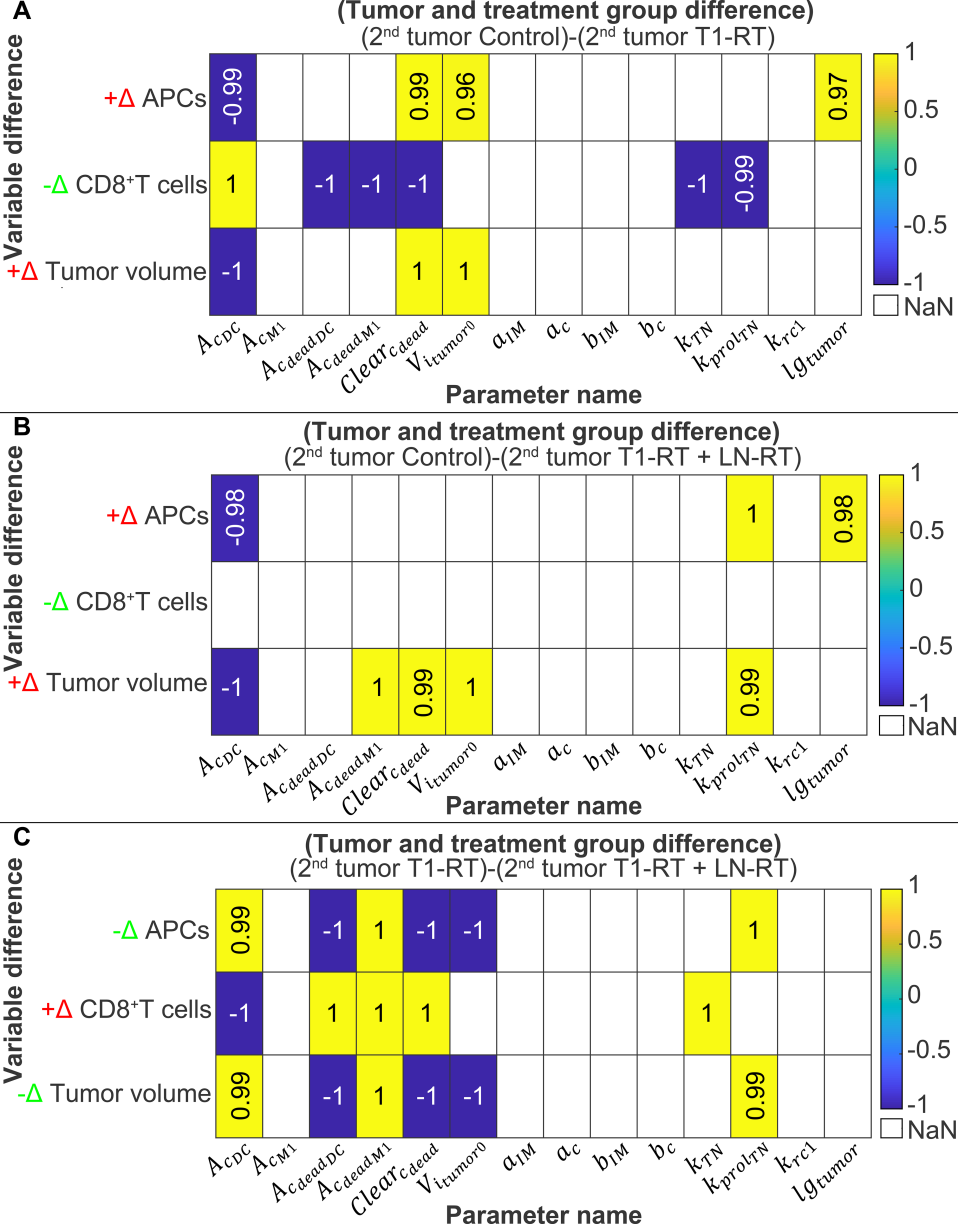
Correlation analysis between model parameter and inter-treatment differences in key immune and tumor variables within the 2^nd^ tumor (T2). Spearman’s rank correlation coefficients (ρ) were computed between one-at-a-time parameter perturbations and time-averaged differences in predicted antigen-presenting cells (APCs), CD8^+^ T cells, and tumor volume among treatment groups. Panels show correlations for differences **(A)** Control vs T1-RT, **(B)** Control vs T1-RT + LN-RT, and **(C)** T1-RT vs T1-RT + LN-RT. The sign indicator (+Δ or −Δ) denotes the direction of the mean difference between groups (e.g., +Δ indicates higher values in the first group listed). E.g., for Control and T1-RT, a positive sign (+Δ) means the variable is higher in Control than T1-RT, and a negative sign (-Δ) vice versa. Rows correspond to model variables and columns to model parameters. Only statistically significant correlations (p< 0.05) that were consistent across bootstrap refits are displayed, i.e., only those averaged ρ values that had the same sign across bootstraps and were statistically significant (p< 0.05) in the majority of the bootstrap replicates (threshold: ≥70% of bootstrap refits) were presented. Positive correlations (yellow) indicate that increasing the parameter enhances the difference between treatment groups, whereas negative correlations (blue) indicate that increasing the parameter reduces the difference. For variables with +Δ difference, positive correlations (yellow) indicate that increasing the parameter enhances the difference between treatment groups, whereas negative correlations (blue) indicate that increasing the parameter reduces the difference. For variables with -Δ difference, positive correlations (yellow) indicate that increasing the parameter reduces the difference between treatment groups, whereas negative correlations (blue) indicate that increasing the parameter enhances the difference.


To quantify how individual parameters drive differences between treatment groups, we performed a Spearman’s rank correlation analysis relating one-at-a-time parameter perturbations to time-averaged inter-group differences in key model outputs (APCs, CD8^+^ T cells, tumor volume) for the local (T1) and distant (T2) tumors (**Figures 6**, **7**). For each bootstrap baseline b=1…B and for each parameter sweep level i, we first computed the time-averaged difference between two groups $g_{1}$and $g_{2}$:

$$\Delta V_{g1-g2,i}^{\left(b\right)}=1/T\int_{0}^{T}\left(V_{g1,i}^{\left(b\right)}\left(t\right)-V_{g2,i}^{b}\left(t\right)\right)dt$$

where $V_{g,i}^{\left(b\right)}\left(t\right)$ denotes the model output at time t obtained when the i-th value of the tested parameter is used together with baseline b. For each bootstrap b we then computed Spearman’s rank correlation coefficient ρ (and associated p-value) between the vector ${\left\{\text{Δ}V_{g_{1}-g_{2},i}^{\left(b\right)}\right\}}_{i}$ and the corresponding vector of parameter levels $\{p_{i}{\}}_{i}$. This yields a distribution of ρ and p-values across bootstrap baselines that reflects how robust a parameter’s effect is to baseline uncertainty. To report stable, baseline-independent correlations, we averaged only those ρ values that had the same sign across bootstraps and were statistically significant at p<0.05 in the majority of bootstraps (threshold: ≥70% of B). The average ρ coefficients that pass the significance and stability criteria are displayed in **Figures 6**, **7**. This procedure identifies correlations that are both statistically significant and consistent in direction across fitted baselines, while filtering out spurious or baseline-dependent associations.

Comparing the Control and T1-RT groups (**Figure 6A**) revealed distinct parameter dependencies for both immune and tumor responses. The difference in APCs (Control − T1-RT) is positive, indicating higher APCs in Control than in T1-RT; this difference correlated negatively with the phagocytic potentials $A_{c_{DC}}$, and positively with clearance rate of dead cancer cells debris $Clear_{c_{dead}}$, initial tumor volume $V_{i_{tumor0}}$, naïve T-cell proliferation constant in lymph nodes $k_{prol_{TN}}$, and tumor growth rate $lg_{tumor}$. Interpreted these in light of the positive sign, the negative correlations imply that increasing phagocytic activity tends to reduce the APC gap (i.e., it raises APC levels in the irradiated condition relative to Control), whereas the positive correlations with $Clear_{c_{dead}}$, $V_{i_{tumor0}}$, $k_{prol_{TN}}$, and $lg_{tumor}$ indicate that greater tumor debris clearance, initial tumor volume, tumor growth, and naïve T-cells proliferation widens the gap either by removing tumor antigen and debris or by producing more antigen due to higher initial volume and tumor growth. For CD8^+^ T cells the difference (Control − T1-RT) is negative, i.e., CD8^+^ levels are higher in T1-RT. Negative correlations of this difference with $Clear_{c_{dead}}$, $A_{c_{dead_{DC}}}$, $k_{TN}$, and $k_{prol_{TN}}$ indicate that faster debris clearance, higher dead-cell phagocytosis, and enhanced naïve-T activation/proliferation increase the (negative) gap, consistent with these processes boosting CD8^+^ T cell expansion after RT. Although $Clear_{c_{dead}}$ does not directly stimulate antigen presentation, its negative correlation with the CD8^+^ difference may reflect an indirect effect, for example, removal of abundant post-RT debris reduces tumor volume which can concentrate and increase the measured T cell density (i.e., same or similar number of T cells in smaller volume can results in higher density). The positive correlations of CD8^+^ T cells in the Control − T1-RT groups with $A_{c_{DC}}$ imply that greater phagocytic rate of viable cancer cells, which generate more antigen presentation pre-irradiation, decrease the difference in CD8^+^ T cell levels in T1-RT group relative to the Control group. The tumor-volume difference (Control − T1-RT, positive because tumors are smaller after RT) correlates negatively with $A_{c_{DC}}$ and positively with $Clear_{c_{dead}}$, $V_{i_{tumor0}}$ and $k_{prol_{TN}}$, mirroring the APC pattern.

Comparing Control with T1-RT + LN-RT (**Figure 6B**) shows broadly similar trends. The APC difference (Control − T1-RT + LN-RT, positive) correlated negatively with $A_{c_{DC}}$ and positively with $Clear_{c_{dead}}$ and $V_{i_{tumor0}}$. These relationships suggest that fast phagocytosis reduces the APC disparity by promoting tumor elimination, while higher initial volume and clearance of dead cancer cells widen the APC gap by increasing the available antigen or removing the dead cancer cells generated by radiotherapy, thus reducing the activation of APCs in T1-RT group. For CD8^+^ T cells, the difference (Control − T1-RT + LN-RT, negative) correlated negatively with $A_{c_{dead_{DC}}}$, $A_{c_{dead_{M1}}}$, $Clear_{c_{dead}}$, $k_{TN}$, and $k_{prol_{TN}}$, and positively with $A_{c_{DC}}$, indicating enhanced dead-cell handling and stronger naïve-T activation/proliferation favor CD8^+^ T cells in T1-RT + LN-RT compared to Control, similarly to **Figure 6A**. The correlations of tumor volume difference in Control vs T1-RT + LN-RT groups indicates that increased phagocytic activity by DC and APC ( $A_{c_{DC}}$) and M1 ( $A_{c_{dead_{M1}}}$) reduces the apparent benefit of RT (making the difference smaller), whereas higher initial tumor volume and clearance of dead cancer cells increase RT’s differential impact on irradiated tumor (T1) when lymph nodes are also irradiated.

The T1-RT versus T1-RT + LN-RT comparison (**Figure 6C**) isolates the effects of nodal irradiation. Here the APC difference is negative (APCs higher in T1-RT + LN-RT) due to the higher tumor volume in T1-RT + LN-RT group, and correlated negatively with $A_{c_{dead_{DC}}}$, $Clear_{c_{dead}}$, and $V_{i_{tumor0}}$, and positively with $A_{c_{dead_{M1}}}$ and $k_{prol_{TN}}$. This pattern suggests that while rapid DC-mediated clearance reduces APC persistence, M1-mediated debris handling and recovery of naïve T cells mitigate APC loss when nodes are irradiated. The CD8^+^ difference is positive (higher CD8^+^ in T1-RT + LN-RT) and correlates negative for $A_{c_{DC}}$ and $k_{prol_{TN}}$, but positive for $A_{c_{dead_{DC}}}$, $A_{c_{dead_{M1}}}$, $Clear_{c_{dead}}$, and $k_{TN}$. This indicates that excessive phagocytic activity by DCs can eliminate viable cancer cells and decrease the difference of CD8^+^ T cells between T1-RT and T1-RT + LN-RT groups, thus making the effect of T cells, lymph node irradiation and their recovery insignificant. Furthermore, increased recovery of naïve T cells in lymph nodes can mitigate the effect of lymph node irradiation. The increased phagocytosis of dead cancer cells by DC and M1 also sustained antigen stimulation in the T1-RT group, and results in higher levels of CD8^+^ T cells in T1-RT relative to T1-RT + LN-RT. The tumor-volume difference is negative (smaller tumors in T1-RT), and has positive associations with $A_{c_{DC}}$, $A_{c_{dead_{DC}}}$, $A_{c_{dead_{M1}}}$, and $k_{prol_{TN}}$ suggest that enhanced phagocytosis of viable and dead cancer cells and naïve T-cell proliferation can reduce the difference in tumor volume between T1-RT and T1-RT + LN-RT groups and make the effect of lymph node irradiation insignificant while higher initial tumor volume has the opposite effect due to the negative correlation.

The correlations observed in the first tumor are mirrored in the second, non-irradiated tumor (**Figure 7**), consistent with a systemic immune mechanism. For Control versus T1-RT in T2 (**Figure 7A**), the APC difference (positive) correlated negatively with $A_{c_{DC}}$, and positive with $Clear_{c_{dead}}$, $V_{i_{tumor0}}$, and $lg_{tumor}$ showing that enhanced phagocytosis reduce APC difference in the distant tumor in the irradiated condition relative to Control while higher initial volume and tumor growth rate increase the difference in APCs by producing more antigen. The CD8^+^ difference in T2 (negative) correlated negatively with $A_{c_{dead_{DC}}}$, $A_{c_{dead_{M1}}}$, $Clear_{c_{dead}}$, $k_{TN}$ and $k_{prol_{TN}}$ and positively with $A_{c_{DC}}$, paralleling the first tumor and indicating that debris handling, CD8^+^ T cell activation and naïve T cells proliferation in lymph nodes shape systemic T-cell priming. Tumor-volume differences in T2 (positive) correlated negatively with $A_{c_{DC}}$ and positively with $Clear_{c_{dead}}$ and $V_{i_{tumor0}}$.

Comparing Control with T1-RT + LN-RT in T2 (**Figure 7B**), the APC difference (positive) correlated negatively with $A_{c_{DC}}$ and positively with $k_{prol_{TN}}$ and $lg_{tumor}$. The tumor-volume differences in this comparison correlated negatively with $A_{c_{DC}}$ and positively with $A_{c_{dead_{M1}}}$, $Clear_{c_{dead}}$, $V_{i_{tumor0}}$ and $k_{prol_{TN}}$. Thus, as in T1, lymph-node irradiation increases the importance of naïve-T-cell regenerative capacity for systemic antigen priming and abscopal effect. Finally, the T1-RT versus T1-RT + LN-RT analysis in T2 (**Figure 7C**) reproduces the T1 findings with some differences: APC differences correlated negatively with $A_{c_{dead_{DC}}}$, $Clear_{c_{dead}}$ and $V_{i_{tumor0}}$ and positively with $A_{c_{DC}}$, $A_{c_{dead_{M1}}}$, and $k_{prol_{TN}}$; CD8^+^ differences correlated negatively with $A_{c_{DC}}$, and positively with $A_{c_{dead_{DC}}}$, $A_{c_{dead_{M1}}}$, $Clear_{c_{dead}}$, and $k_{TN}$; and tumor-volume differences correlated negatively with $A_{c_{dead_{DC}}}$, $Clear_{c_{dead}}$, and $V_{i_{tumor0}}$, and positively with $A_{c_{DC}}$, $A_{c_{dead_{M1}}}$, and $k_{prol_{TN}}$. These results again highlight that when lymph nodes are irradiated, phagocytic rates and naïve-T-cell recovery become principal determinants of distant tumor control.

In summary, **Figures 6**, **7** show a coherent mechanistic picture: phagocytic parameters ( $A_{c_{DC}}$, $A_{c_{dead_{DC}}}$, $A_{c_{dead_{M1}}}$) and debris clearance ( $Clear_{c_{dead}}$) determine antigen persistence and hence the strength and duration of APC- and T-cell stimulation, initial tumor volume and tumor growth rate affects the amount of tumor antigen and thus the systemic immune response and naïve-T-cell proliferation ( $k_{prol_{TN}}$) becomes critical when lymphoid tissue is compromised by radiation. The sign of each intergroup difference must be considered when interpreting correlation directions because a positive ρ can mean opposite biological effects depending on whether the first group has higher or lower absolute values.

## 4 Discussion

Our PBPK–QSP modeling analysis offers a coherent mechanistic explanation for when and why localized radiotherapy produces systemic (abscopal) responses and how those responses depend on antigen amount, antigen handling, T-cell dynamics, and lymphoid integrity. The core message from **Figures 6**, **7** is that parameters that control antigen production (initial tumor volume $V_{i_{tumor0}}$ and tumor growth rate $lg_{tumor}$), antigen capture and processing ( $A_{c_{DC}}$, $A_{c_{dead_{DC}}}$, $A_{c_{dead_{M1}}}$), clearance of dead cancer cells debris ( $Clear_{c_{dead}}$), and lymphoid regenerative capacity ( $k_{prol_{TN}}$) exert the largest and most interpretable influences on both local (T1) and distant (T2) tumors responses.

These model results align with key mechanistic and translational findings in literature. The requirement for radiation-driven antigen release and subsequent DC-dependent cross-priming to generate systemic CD8^+^ responses is well established in preclinical studies and clinical case reports (for example, radiation plus CTLA-4 in mouse models (5) and human ipilimumab and radiotherapy case report (9)). Fractionation and dose per fraction are critical determinants of immunogenicity: preclinical work showed that fractionated radiotherapy synergizes with anti-CTLA-4 to induce abscopal responses whereas single large doses may not (5), and more recent work identified TREX1 induction as a dose-dependent limiter of cytosolic DNA–STING signaling and immunogenicity (7). The central role of immunogenic cell death (release of TAAs) and DC activation in converting local tumor ablation into systemic T-cell responses is supported by foundational studies on HMGB1/TLR4 signaling (43) and the broader immunogenic-cell-death literature (44); our model’s sensitivity to phagocytic parameters ( $A_{c_{DC}}$, $A_{c_{dead_{DC}}}$) and to APC dynamics recapitulates these mechanistic dependencies. When phagocytosis and debris clearance are too rapid, antigen exposure is shortened and the window for DC priming narrows; conversely, inefficient clearance prolongs antigen exposure but can also create immunosuppressive contexts, which explains why the net effect of phagocytic parameters in our correlations is context dependent.

Clinically, our model’s implications resonate with trials and case series showing that adding immune stimulation to radiotherapy (e.g., GM-CSF, checkpoint blockade) increases the probability of observable abscopal responses, but that design details matter (3). Golden et al. trial with GM-CSF and radiotherapy as well as other clinical reports indicate that enhancing DC maturation or reducing immune checkpoints can convert sporadic abscopal events into reproducible responses (45). All these findings are consistent with ours in that improving antigen presentation (increasing effective $k_{TN}$) or preserving T-cell regenerative capacity ( $k_{prol_{TN}}$) increases systemic T-cell levels and distant tumor control.

A particularly robust and clinically relevant insight from our analysis concerns the impact of lymph-node irradiation. Multiple preclinical studies have shown that tumor-draining lymph nodes (TDLNs) are essential hubs for T-cell priming and for generating a stem-like progenitor pool that feeds effector responses (46–48); elective or incidental irradiation of DLNs can attenuate chemokine expression, diminish stem-like CD8^+^ populations, and blunt the abscopal benefit of tumor-directed stereotactic radiotherapy (35, 49). Our correlations show that when lymph nodes are irradiated (T1-RT + LN-RT), parameters governing lymphoid regeneration ( $k_{prol_{TN}}$) gain prominence, which is exactly the pattern reported by Darragh et al. (50) and Buchwald et al. (35), who found that lymph node irradiation attenuates the combinatorial benefit of radiotherapy and ICB. These concordant findings argue that careful radiotherapy field design (sparing or delaying draining lymph node irradiation wherever feasible) and strategies to protect or restore lymphoid function may be required to maximize systemic antitumor immunity.

From these mechanistic and translational comparisons, we derive three practical, model-driven recommendations. First, optimize radiotherapy dose/fractionation to maximize immunogenic antigen release without inducing dose-dependent suppressors of immunity (e.g., TREX1-mediated loss of cGAS–STING signaling) and avoid unnecessary irradiation of lymph node when clinical circumstances permit. Second, combine radiotherapy with interventions that either (a) prolong effective antigen presentation (DC maturation, timed GM-CSF or TLR agonists), (b) modulate phagocytic clearance kinetics to preserve a productive antigen exposure window, or (c) protect and/or accelerate lymphoid recovery (cytokine support, timing of ICI relative to radiotherapy).

The model necessarily simplifies complex spatial and cellular heterogeneity by treating compartments as well-mixed and by lumping antigenic states and several myeloid/lymphoid subpopulations into aggregate variables. We deliberately parameterized extravasation and vascular permeability to reflect abnormal tumor vasculature and we do not consider any effect of radiotherapy on vasculature (51); this choice explains the modest differences in intratumoral CD8^+^ densities between T1 and T2 and highlights that trafficking constraints can limit the translation of systemic activation into local infiltration. Another important limitation comes from the calibration data, which are derived from bilateral subcutaneous flank melanoma tumors. This subcutaneous model might not fully recapitulate the native melanoma microenvironment, which might alter lymphatic drainage, immune-cell trafficking, and local immune regulation. This biological distinction may therefore affect the generalizability of some predictions, particularly those that depend on local stromal effects. Future work should couple our PBPK–QSP core to spatially resolved tumor models that capture heterogeneous dose distributions, intratumoral microenvironmental gradients, and explicit lymphatic anatomy, so that predictions can be extended to partial-volume irradiation, cold-spot scenarios, and clinically realistic dose-painting strategies. A pragmatic, intermediate extension is to compartmentalize each tumor into perfused and non-perfused (hypoxic) subregions, allowing time-dependent changes in the perfused fraction or permeability to approximate vascular remodeling and its effect on immune infiltration without requiring a full spatial discretization. Such a compartmental approach would need less data for parameterization and validation than full spatio-temporal models and could directly capture clinically relevant scenarios and generate testable hypotheses for prospective preclinical validation.

In conclusion, our integrated PBPK–QSP modeling framework explains how local radiotherapy can produce systemic tumor control and why that outcome is highly parameter dependent: local radiosensitivity, antigen handling and clearance, and lymphoid regenerative capacity jointly determine whether a strong abscopal response emerges or is suppressed. These findings synthesize and extend experimental and clinical observations and provide a quantitative framework to design radiotherapy and immunotherapy that maximize systemic benefit while minimizing collateral damage to critical immune hubs.

## Data Availability

The original contributions presented in the study are included in the article/**Supplementary Material**. The COMSOL Multiphysics model and the optimal sets of parameters used to generate the data for this study can be found on Zenodo: https://doi.org/10.5281/zenodo.18624161. Further inquiries can be directed to the corresponding author.
