# Supplementary material for: Mechanistic mathematical modeling of abscopal effect reveals mechanisms of off-target tumor response
Source: Front Immunol. 2026 Mar 16;17:1769229. doi: 10.3389/fimmu.2026.1769229 (PMC13033604; doi:10.3389/fimmu.2026.1769229)
Supplement: Supplementary file 1 [file DataSheet1.pdf]

# *Supplementary Material*

## 1 EQUATIONS:

The physiologically based pharmacokinetics (PBPK) model was utilized to simulate the dynamic behavior and distribution of various immune cell populations across multiple organs (compartments). The model includes 10 different cell types: dendritic cells (DCs), macrophages type 1 (M1) and type 2 (M2), regulatory T cells (Tregs), viable and dead tumor cells (Tv and Td respectively) and naïve $CD8^{+}$ T (TN) and tumor specific CD8+ effector T cells (TE1), and antigen-presenting cells (APCs). The model tracks seven out of ten cell types (all excluding TN, Tv and Td) as they are circulating throughout 12 key organs/compartments, including: lungs, liver, gastrointestinal tract, spleen, heart, kidneys, skin, muscle, bone, lymph nodes, and 2 tumors. TN cells are calculated only in the lymph nodes where they exist in large quantities, and similarly, Tv and Td are calculated only in the tumor compartments.

The aforementioned 12 compartments were divided into two sub-compartments: the vascular and extravascular spaces. Beyond these compartments, an extra compartment for blood circulation was added, and it did not divide into sub-compartments. For blood circulation and each sub-compartment, population conservation equations were formulated to capture the dynamics of every cell type. These equations describe the balance of cell populations, number of cells per volume, in terms of their accumulation in the volume of the compartment or sub-compartment, expressed as: cell accumulation = incoming fluxes - outgoing fluxes + transport across sub-compartments (vascular to extravascular space and vice versa) + other process such as cellular proliferation, death, cytolysis, phagocytosis, activation against tumor antigen, and deactivation of anti-tumor immune cells induced by regulatory cells (Tregs and M2).

The equations for the transport of dendritic cells through the vascular and extravascular space are presented below, and similar equations are also used for the other cells, with some extra terms which are presented later in Supplementary Table 1.

The free circulating dendritic cells ($DC_{v_{j_F}}$, cells per volume) in the vascular space of the j-th compartment are given as per the equation (1) below:

$$V_{v_j} * \frac{dDC_{v_{j_F}}}{dt} = \sum_{\substack{k=organs \\ k\neq j,\ lnode}} \left( Q_k^{in} DC_{v_{k_F}}^{in} - Q_k^{out} DC_{v_{j_F}} \right) - J_{DC\,j} * \left(1 - \frac{Im_{i_j}}{IM_{i_{max\,j}}}\right) * DC_{v_{j_A}} * V_{v\,j} \quad (1)$$

where the first term on the left side is the accumulation of $DC_{v_{j_F}}$ in the volume of the vascular space ($V_{v_j}$), the sum on the right side is the incoming minus the outgoing fluxes, where $Q_k^{in}$ is the blood

flow from the k-th compartment and $Q_k^{out}$ is the outgoing blood flow from the j-th to the k-th compartment, which is multiplied by $DC_{v_{k_F}}^{in}$ (number of DC per volume in the k-th compartment) and $DC_{v_{j_F}}$ (number of DC per volume in the j-th compartment) respectively. The second term is the transmigration of DC from the vascular to the extravascular space, which depends on the transmigration constant ($J_{DC\,j}$) and the level of immune cell saturation in the extravascular space of the j-th compartment, where $Im_{i\,j}$ is the amount of immune cells and $IM_{i_{max\,j}}$ is the maximum immune cells in the extravascular space of the j-th compartment.

The dendritic cells in the extravascular space ($DC_{i\,j}$, cells per volume) of the j-th compartment are given as per the equation (2) below:

$$V_{i\,j} * \frac{dDC_{i\,j}}{dt} = J_{DC\,j} * \left(1 - \frac{Im_{i\,j}}{Im_{i_{max\,j}}}\right) * DC_{v_{j_A}} * V_{v\,j} - L_j * delta_{DC\,j} * \left(1 - \frac{Im_{i_{lnode}}}{Im_{i_{max\,lnode}}}\right) * DC_{i\,j} + phi_{DC\,j} * DC_{i\,j} * \left(1 - \frac{Im_{i\,j}}{Im_{i_{max\,j}}}\right) * V_{i\,j} \quad (2)$$

where the first term on the left side is the accumulation of $DC_{i\,j}$ in the volume of the extravascular space ($V_{i\,j}$), on the right side, the first term is the transmigration of dendritic cells from the vascular to extravascular space, the second term is migration to lymph nodes via lymphatic vessels, and the last term is the proliferation of $DC_{i\,j}$. The transmigration term is the same as the equation (1) But with a positive sign in the equation (2). The migration to lymph nodes depends on the fluid absorption rate by the lymphatic vessels ($L_j$), the fraction ($delta_{DC\,j}$) of $DC_{i\,j}$ that can be recirculated, and the level of immune cell saturation in the lymph nodes. The proliferation term depends on the proliferation rate constant $phi_{DC\,j}$ and the level of immune cell saturation in the extravascular space.

The dendritic cells in the extravascular space ($DC_{i_{lnode}}$, cells per volume) of the lymph node compartment are given as per the equation (3) below:

$$V_{i_{lnode}} * \frac{dDC_{i_{lnode}}}{dt} = J_{DC_{lnode}} * \left(1 - \frac{Im_{i_{lnode}}}{Im_{i_{max_{lnode}}}}\right) * DC_{v_{lnode_A}} * V_{v_{lnode}} + \sum_{k=organs} \left[L_k * delta_{DC_k} \left(1 - \frac{Im_{i_{lnode}}}{Im_{i_{max_{lnode}}}}\right) * DC_{i_k}\right] + phi_{DC_{lnode}} * DC_{i_{lnode}} * \left(1 - \frac{Im_{i_{lnode}}}{Im_{i_{max_{lnode}}}}\right) * V_{i_{lnode}} \quad (3)$$

where the first term on the left side is the accumulation of $DC_{i_{lnode}}$ in the volume of the extravascular space ($V_{i_{lnode}}$), on the right side, the first term is the transmigration of dendritic cells from the vascular to the extravascular space, the second term is the recirculation of $DC_{i_k}$ from the k-th compartments to the lymph node, and the last term is the proliferation of $DC_{i_{lnode}}$. The first and last terms on the right side of the equation (3) are similar to the equation (2) but the second term differs to consider the recirculation of dendritic cells from all compartments back to the lymph nodes.

The free dendritic cells ($DC_{v_{blood_F}}$, cells per volume) in the blood circulation are given as per the equation (4) below:

$$V_{v_{blood}} * \frac{dDC_{v_{blood_F}}}{dt} = \sum_{k=organs} \left(Q_k^{in} DC_{v_{k_F}}^{in} - Q_k^{out} DC_{v_{j_F}}\right) \quad (4)$$

where the first term on the left side is the accumulation of $DC_{v_{blood_F}}$ in the volume of the blood circulation ($V_{v_{blood}}$), and the sum on the right side is the incoming minus the outgoing fluxes, where $Q_k^{in}$ is the blood flow from the k-th compartment and $Q_k^{out}$ is the outgoing blood flow from the blood compartment to the k-th compartment, which is multiplied by $DC_{v_{k_F}}^{in}$ (number of DC per volume in the k-th compartment) and $DC_{v_{j_F}}$ (number of DC per volume in the j-th compartment) respectively.

In the PBPK model (equations 1-4), additional terms were incorporated into the population conservation equations for specific immune cell types and compartments to account for specific biological interactions. These terms reflect critical processes such as cell-to-cell interactions within the tumor microenvironment and other compartments. The terms that differentiate equations 1-4 for the different types of immune cells are presented in Supplementary Table 1.

**Supplementary Table 1: Extra terms for the equations 1-4 for specific immune cell types and compartments to account for specific biological interactions.**

| Number of the equation where the term is added | Immune cell type for which the equation changed | j-th compartment where the term has effect | Extra term for the equations 1-6 for this immune cell and compartment |
|---|---|---|---|
| (1) | $APC_{v_{j_F}}$ | Bone, heart, kidney, liver, lung, muscle, skin, spleen, gastrointestinal tract, lymph nodes, tumor | $-(k_{sup_{M2_{APC}}} * M2_{v_{j_F}} + k_{sup_{Treg_{APC}}} * Treg_{v_{j_F}}) * APC_{v_{j_F}} * V_{v_j}$ |
| (2) | $APC_{i_j}$ | Bone, heart, kidney, liver, lung, muscle, skin, spleen, gastrointestinal tract, tumor | $-(k_{sup_{M2_{APC}}} * M2_{i_j} + k_{sup_{Treg_{APC}}} * Treg_{i_j}) * APC_{i_j} * V_{i_j}$ |
| (2) | $APC_{i_j}$ | Tumor | $+ x_{DC} * A_{c_{DC}} * \left(C_{i_{tumor}} + C_{i_{tumor_{dead}}}\right) * DC_{i_j} * V_{i_j} + x_{M1} * A_{c_{M1}} * (C_{i_{tumor}} + C_{i_{tumor_{dead}}}) * M1_{i_j} * V_{i_j}$ |
| (2) | $APC_{i_j}$ | Lymph nodes | $\begin{cases} -k_{radio_{Im}} * APC_{i_j} * V_{i_j}, for\ t_r \\ 0, otherwise \end{cases}$ |
| (2) | $DC_{i_j}$ | Tumor | $- x_{DC} * A_{c_{DC}} * (C_{i_{tumor}} + C_{i_{tumor_{dead}}}) * DC_{i_j} * V_{i_{tumor}}$ |

| | | | |
|---|---|---|---|
| (2) | $M1_{i_j}$ | Tumor | $-x_{M1} * A_{c_{M1}} * (C_{i_{tumor}} + C_{i_{tumor_{dead}}}) * M1_{i_j} * V_{i_j} - x_{M1_{to_{M2}}} * C_{i_{tumor}} * M1_{i_j} * V_{i_j}$ |
| (2) | $M2_{i_j}$ | Tumor | $+x_{M1_{to_{M2}}} * C_{i_{tumor}} * M1_{i_j} * V_{i_j}$ |
| (1) | $TE1_{v_{j_F}}$ | Bone, heart, kidney, liver, lung, muscle, skin, spleen, gastrointestinal tract, lymph nodes, tumor | $-(k_{sup_{M2_{TE1}}} * M2_{v_{j_F}} + k_{sup_{Treg_{TE1}}} * Treg_{v_{j_F}}) * TE1_{v_{j_F}} * V_{v_j}$ |
| (2) | $TE1_{i_j}$ | Bone, heart, kidney, liver, lung, muscle, skin, spleen, gastrointestinal tract, tumor | $-(k_{sup_{M2_{TE1}}} * M2_{i_j} + k_{sup_{Treg_{TE1}}} * Treg_{i_j}) * TE1_{i_j} * V_{i_j}$ |
| (2) | $TE1_{i_j}$ | Lymph nodes | $+k_{TN} * TN_{i_j} * APC_{i_j} * V_{i_j}$ <br> $\begin{cases} -k_{radio_{Im}} * TE1_{i_j} * V_{i_j}, for\ t_r \\ 0, otherwise \end{cases}$ |
| $k_{radio_{Im}} = \frac{1}{\Delta\tau} * \left(1 - exp(-a_{Im} * D_{radio} - b_{Im} * D_{radio}^2)\right)$ | | | |

Supplementary Table 1 includes the suppression of APCs and TE1 by M2 Macrophages and Tregs. For antigen-presenting cells (APCs) and CD8+ effector T cells (TE1), suppression terms were introduced across multiple organs in both extravascular spaces and vascular spaces (see Supplementary Table 1). These terms account for the suppressive effects of M2 macrophages and regulatory T cells (Tregs) on the respective immune cell populations (e.g. $-(k_{sup_{M2_{APC\ or\ TE1}}} * M2_{i_j\ or\ v_j} + k_{sup_{Treg_{APC\ or\ TE1}}} * Treg_{i_j\ or\ v_j}) * Cell_{i_j\ or\ v_j} * V_{i_j\ or\ v_j}$). These terms reduce the populations of APCs and TE1 in compartments where M2 macrophages and Tregs exert suppression.

Another term in Supplementary Table 1 is related to tumor-specific activation of APC. Within the tumor compartment, an additional activation term was added for APCs in the extravascular space of the tumor compartment. This term accounts for the activation of APCs from dendritic cells (DCs) and

M1 macrophages in response to tumor-associated antigens after phagocytosing both viable and dead tumor cells (e.g. $+x_{M1} * A_{c_{DC\,or\,M1}} * (C_{i_{tumor}} + C_{i_{tumor_{dead}}}) * Cell_{i_j} * V_{i_j}$). This term reflects the activation of APCs at the tumor site in response to immune-stimulatory signals and is added to the equation of APC and subtracted from the equation of DC or M1. An extra term is added in Lymph node for APCs and TE1 to account the effect of lymph nodes irradiation ($-k_{radio_{Im}} * TE1_{i_j} * V_{i_j}$), this term is activated only at the time of radiotherapy ($t_r$). Furthermore, M1 macrophages can transition into M2 macrophages under tumor-induced polarization with the term $x_{M1_{to_{M2}}} * C_{i_{tumor}} * M1_{i_j} * V_{i_j}$ which is added to the equation of M2 and subtracted from the equation of M1.

The last extra term is related to the activation of CD8+ effector T cells (TE1) in lymph nodes. In the lymph node compartment, an additional term was added to the extravascular TE1 equation to reflect the activation of naive T cells ($TN_{i_j}$) by APCs, $k_{TN} * TN_{i_j} * APC_{i_j} * V_{i_j}$ and population balance of naive T cells in lymph nodes is given by the following equation where the proliferation of naïve T cells, activation by APCs, and the effect of lymph node irradiation is considered.

$$V_{i_{lnode}} * \frac{dTN_{i_{lnode}}}{dt} = \left(k_{prol_{TN}} * TN_{i_{lnode}} * \left(1 - \frac{TE1_{i_{lnode}} + TN_{i_{lnode}}}{TN0_{i_{lnode}}}\right) - k_{TN} * TN_{i_{lnode}} * APC_{i_{lnode}} - \begin{cases} k_{radio_{Im}} * TN_{i_{lnode}}, for\ t_r \\ 0, otherwise \end{cases}\right) * V_{i_{lnode}} \quad (5)$$

## 2 Population conservation of cells in the tumor compartments

The population conservation equations of cells in the tumor compartments have the same formulation as described previously in equations 1-2, with the only difference on the left side of the equation (the accumulation) where the total volume and the density or concentration (number of cells per volume) of the cells changed simultaneously. For this reason, the accumulation was calculated for all cells based on their total number in the tumor compartment ($\frac{d\,(DC_{i_{tumor}} * V_{i_{tumor}})}{dt}$ or $\frac{d\,(DC_{v_{tumor}} * V_{v_{tumor}})}{dt}$ where $DC_{i_{tumor}} * V_{i_{tumor}}$ is the total number of DC in the tumor $n_{i_{tumor}}^{DC}$) and the density or concentration (cells per volume) of each cell was calculated by dividing the total number with the current tumor volume ($\frac{n_{i_{tumor}}^{DC}}{V_{i_{tumor}}}$).

The viable and dead tumor cells were calculated only in the extravascular space in the tumor compartment. The number of viable tumor cells in the extravascular space ($n_{i_{tumor}}^{C}$, total number of viable tumor cells) of the tumor compartment are given as per the equation (6) below:

$$\frac{dn^{c}_{i_{tumor}}}{dt} = \left( lg_{tumor} * C_{i_{tumor}} * \left(1 - \frac{Cell_{i_{tumor}}}{C_{tumor_{max}}}\right) - A_{c_{DC}} * C_{i_{tumor}} * \left(DC_{i_{tumor}} + APC_{i_{tumor}}\right) - A_{c_{M1}} * C_{i_{tumor}} * M1_{i_{tumor}} - C_{i_{tumor}} * k_{rc1} * TE1_{i_{tumor}} - \begin{cases} k_{radio_c} * C_{i_{tumor}}, for\ t_r \\ 0, otherwise \end{cases} \right) * V_{i_{tumor}} \quad (6)$$

where the first term is the tumor cell proliferation which is proportional to the growth rate constant $lg_{tumor}$ and the density of viable cancer cells $C_{i_{tumor}}$ (cells per volume) and restricted with the levels of cells in extravascular space of the tumor, the second term is the phagocytosis of cancer cells by dendritic cells (DCs) and antigen-presenting cells (APCs), and the following term is phagocytosis of cancer cells by the macrophages (M1), the following term is the cytolysis of cancer cells by cytotoxic effects of CD8+ effector T cells (TE1) and the last term is the effect of radiotherapy where $k_{radio_c} = \frac{1}{\Delta\tau} * \left(1 - exp(-a_c * D_{radio} - b_c * D_{radio}^2)\right)$ and is calculated only at the time of the radiotherapy $(t_r)$.

The number of dead tumor cells in the extravascular space ($n^{D}_{i_{tumor}}$, total number of dead tumor cells) of the tumor compartment are given as per the equation (7) below:

$$\frac{dn^{d}_{i_{tumor}}}{dt} = \left(-A_{c_{DC}} * D_{i_{tumor}} * \left(DC_{i_{tumor}} + APC_{i_{tumor}}\right) - A_{c_{M1}} * D_{i_{tumor}} * M1_{i_{tumor}} + C_{i_{tumor}} * k_{rc1} * TE1_{i_{tumor}}\right) * V_{i_{tumor}} \quad (7)$$

where the first and second terms are the phagocytosis of dead cancer cells ($D_{i_{tumor}}$, cells per volume) by DC, APC, and M1 macrophages, and the last term is tumor cell death induced by effector CD8+ T cells (TE1).

The tumor volume of the extravascular space $V_{i_{tumor}}$ was calculated based on the change in the total number of all cells in the extravascular space and the current density of the cells $Cell_{i_{tumor}}$ (total number of cells per volume) as per the equation (8) below.

$$\frac{dV_{i_{tumor}}}{dt} = \frac{\sum_{q=c,d,DC,APC,TE1,TE2,M1,M2,Treg} \frac{dn^{q}_{i_{tumor}}}{dt}}{Cell_{i_{tumor}}} \quad (8)$$

where the sum of the q-th cell type calculates the change in the total number of all cells in the tumor and the deviation by the current density of the cells ($Cell_{i_{tumor}}$) results in the change of volume ($\frac{dV_{i_{tumor}}}{dt}$).

## 3 Estimation of Model Parameters

The optimization algorithm estimates the following parameters that are related to the tumor cell line, tumor growth rate constant $lg_{tumor}$, the initial tumor volume $V_{i_{tumor}0}$ and clearance rate constant of dead cancer debris ($Clear_{c_{dead}}$), parameters that are related to the immune response, phagocytic rate constant of viable cancer cells by DC and APC ($A_{c_{DC}}$), and by M1 ($A_{c_{M1}}$), phagocytic rate constant of dead cancer cells by DC and APC ($A_{c_{dead_{DC}}}$), and by M1 ($A_{c_{dead_{M1}}}$), proliferation rate constant of naïve CD8$^+$ T cells ($k_{prol_{TN}}$), the activation rate constant of naïve CD8$^+$ T cells $k_{TN}$, and cytolytic rate constant of cancer cells by CD8$^+$ effector T cells $k_{rc1}$, and parameters related to linear–quadratic model for the radiosensitivity, $a_c$ and $b_c$ the 1st and 2nd order component respectively of cancer cells, $a_{IM}$ and $b_{IM}$ the radiosensitivity parameter of APCs and the naïve and effector CD8$^+$ T cells.

From these parameters, 12 parameters remained consistent across treatment groups ($lg_{tumor}$, $V_{i_{tumor}0}$, $Clear_{c_{dead}}$, $A_{c_{DC}}$, $A_{c_{M1}}$, $A_{c_{dead_{DC}}}$, $A_{c_{dead_{M1}}}$, $k_{prol_{TN}}$, $a_c$, $b_c$, $a_{IM}$, $b_{IM}$) while 2 parameters ($k_{rc1}$, $k_{TN}$) varied between radiotherapy and control treatments. The key parameters that were assumed to be affected by radiotherapy are the parameters which control the activation and the cytolytic effect of CD8$^+$ T cells due to the increased tumor antigen by radiotherapy that stimulate the adaptive immune system. Supplementary Table 2 presents the model parameters that were calculated by the optimization process, and Supplementary Figure 1 presents the probability distributions of the fitted parameters calculated from the bootstrap and refit process.

**Supplementary Table 2: Parameters estimated by optimization and are affected by cancer cell type, cancer-immune cell interaction, and radiotherapy (RT).**

| Parameter | Description | Value with peak probability |
|---|---|---|
| $lg_{tumor}$ | Tumor growth rate constant | 0.432 [1/d] |
| $V_{i_{tumor0}}$ | Initial tumor volume | 38.6 [mm$^3$] |
| $Clear_{c_{dead}}$ | Clearance rate constant of cancer debris (dead cancer cells) | 6.77×10$^{-9}$ [d$^{-1}$] |
| $A_{c_{DC}}$ | Phagocytic rate constant by dendritic cells/APCs | 661 [cm$^3$/d] |
| $A_{c_{M1}}$ | Phagocytic rate constant by M1 macrophages | 351 [cm$^3$/d] |
| $A_{c_{dead_{DC}}}$ | Phagocytic rate constant of dead cancer cells by dendritic cells/APCs | 18.3 [cm$^3$/d] |

| | | |
|---|---|---|
| $A_{c_{dead_{M1}}}$ | Phagocytic rate constant of dead cancer cells by M1 macrophages | 99100 [$cm^3/d$] |
| $k_{prol_{TN}}$ | Proliferation rate constant of naïve $CD8^+$ T cells | 84.8 [$d^{-1}$] |
| $a_c$ | Radiosensitivity parameter of cancer cells, $1^{st}$ order component of linear–quadratic model | 4.28 [$Gy^{-1}$] |
| $b_c$ | Radiosensitivity parameter of cancer cells, $2^{nd}$ order component of linear–quadratic model | 0.17 [$Gy^{-2}$] |
| $a_{IM}$ | Radiosensitivity parameter of APCs and $CD8^+$ T cells, $1^{st}$ order component of linear–quadratic model | 3.23 [$Gy^{-1}$] |
| $b_{IM}$ | Radiosensitivity parameter of APCs and $CD8^+$ T cells, $2^{nd}$ order component of linear–quadratic model | 39.7 and 48.3 [$Gy^{-2}$] |
| $k_{TN}$ | Activation rate constant of naïve $CD8^+$ T cells | $3.33\times10^{-5}$ [$cm^3/d$] for control |
| | | $1.68\times10^{-4}$ [$cm^3/d$] for RT |
| $k_{rc1}$ | Cytolytic rate of tumor cells by effector $CD8^+$ T cells | 0.209 [$cm^3/d$] for control |
| | | 0.446 [$cm^3/d$] for RT |

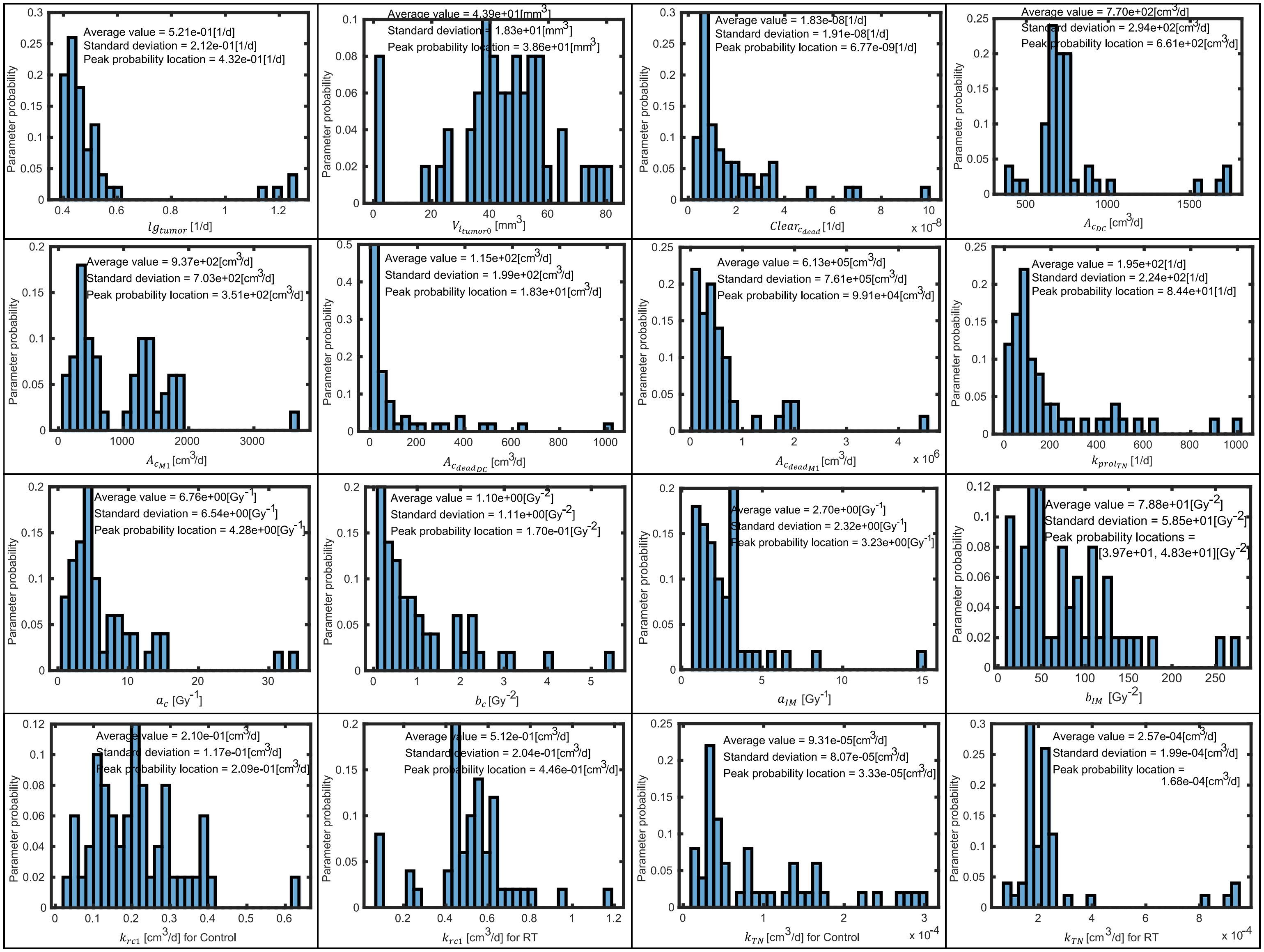

**Supplementary Figure 1: Probability distributions of the fitted parameters from the bootstrap resampling and refit process**

### 3.1 Pseudo algorithm of the bootstrap resampling and refit process

```
Inputs:
  data_table_tr_nt        // experimental table split by Treatment × Tumor
  idx_subjPerTreatPer_n   // cell array of subject IDs per (treatment, tumor)
  n_subjPerTreatPer_n     // number of subjects per (treatment, tumor)
  initialIndividuals0     // initial GA population matrix (rows = individuals, cols = nParam in log-space)
  B                       // number of bootstrap replicates
  nParam                  // number of fitted parameters
  gaOptionsTemplate       // GA options template (PopulationSize, MaxGenerations, ...)
  COMSOL_model_file       // path to .mph model
Output:
  bootResults.mat with params_boot, obj_boot, convergence_flag, etc.

Algorithm:

1. Pre-allocate storage:
   params_boot  = nan(B, nParam)
   obj_boot     = nan(B, 1)
   convergence_flag = false(B,1)

2. For b = 1 : B
     // (your code begins at b=27; generalised here)
   a. Build bootstrap sample (stratified by Treatment × Tumor):
     For tt = each treatment
       For nn = each tumor index
         idxs = idx_subjPerTreatPer_n{tt,nn}
         nsub = n_subjPerTreatPer_n{tt,nn}
         draws = randsample(idxs, nsub, true)   // sample subjects with replacement
         bsSubjects{tt,nn} = draws
         extract rows for each drawn Mouse_ID from data_table_tr_nt{tt,nn}
         res_data_table_tr_nt{tt,nn} = sampled rows in drawn order
     End loop over tumor indexes
     End loop over tumor treatments
```

```
bsData = vertcat(res_data_table_tr_nt{:})   // concatenated bootstrap dataset
```

**b.** Build GA initial population for this replicate:

```
initialPopulation = [ initialIndividuals0 ; params_boot(1:b-1, :) ]
  // include baseline initialIndividuals0 and all previous bootstrap optima to accelerate convergence

// construct adaptive bounds in log-space around the initial population
lb_1 = min(initialPopulation, [], 1)'  -  abs(min(initialPopulation,[],1)') .* (0.1 * rand(nParam,1))
ub_1 = max(initialPopulation, [], 1)'  +  abs(max(initialPopulation,[],1)') .* (0.1 * rand(nParam,1))
// Note: bounds randomized ±10% to avoid trapping at hard limits
```

**c.** Configure GA options (use gaOptionsTemplate, set InitialPopulationMatrix = initialPopulation)

```
gaOptions = set options (PopulationSize, CrossoverFcn, MutationFcn, UseParallel = true, ...)
```

**d.** Define objective function handle for GA:

```
objective_fun_data = @(ln_param) objective_fun(ln_param, bsData)
   // ln_param is parameter vector in log-space. objective_fun should:
   //  - exponentiate ln_param -> param vector (positive)
   //  - run COMSOL simulations for each (Treatment_Tumor) case
   //  - interpolate model outputs at experimental times
   //  - compute squared errors and aggregate into scalar SSE
```

**e.** Run GA:

```
[optim_ln_params, fval, exitflag, output] = ga(objective_fun_data, nParam, lb_1, ub_1, gaOptions)
params_boot(b, :) = optim_ln_params   // store best ln-parameters
obj_boot(b) = fval
convergence_flag(b) = (exitflag > 0)
```

**f.** Periodically save progress:

```
save('bootResults.mat', 'params_boot', 'obj_boot', 'convergence_flag', '-v7.3')
```

**3. End loop over bootstrap replicates**

**4.** Post-processing:
  - exponentiate params_boot to obtain parameter distributions on natural scale
  - compute empirical statistics (mean, sd, percentiles) and save to repository
  - produce diagnostic plots (histograms, KDEs, ensemble model curves)

-------------------------------------------------------

**Function:** objective_fun(ln_param, data_table)
 // ln_param: column vector of length nParam (log-scale parameters)
 **1.** param = exp(ln_param)   // convert to natural scale
 **2.** Determine which model simulations to run:
      - identify unique Treatment_Tumor cases present in data_table
 **3.** Ensure COMSOL model is loaded on each parallel worker:
      if not initialized: load COMSOL model on workers and call sharedModel('initialize', model)
 **4.** For each unique Treatment_Tumor in parallel (parfor):
      **a.** Get worker-specific shared COMSOL model
      **b.** Set model parameters from param:
        - V_tumor0, lg_tumor, A_c_DC, A_c_M1, A_c_dead_DC, A_c_dead_M1,
          Clear_tumor_dead, k_rc1, k_TN, a_Im, b_Im, a_c, b_c, k_prol_TN, ...
        - configure RT/LN flags by setting COMSOL functions rect1/rect2 amplitudes
      **c.** Clear previous solution and run model.study('std1').run()
      **d.** Check solver convergence and collect any warnings; set converged = false if problems occur
      **e.** Using model.result.numerical('gev1'), set interp times = experimental times for this group
      **f.** Extract predicted values at experimental times
      **g.** If converged == false, apply fallback values for times beyond solver final time (e.g., fixed conservative values)
      **h.** Compute squared errors per observation: (data_value - model_pred)^2
      **i.** Aggregate squared errors per variable/time (accumarray) and weight as needed
 **5.** Collect per-group errors across parallel workers
 **6.** Reduce aggregated errors to scalar SSE:
      error_t = mean( per-group mean squared errors )  // or other averaging rule used
 **7.** Return error_t to GA

**Notes / Implementation details:**
 - All optimization is in log-space and COMSOL receives parameters in physical units.
 - The GA runs in parallel; each candidate evaluation launches COMSOL evaluations on workers via sharedModel.

- Non-convergent COMSOL runs are handled gracefully by assigning fallback model outputs to ensure finite SSEs.
- Objective uses tumor volumes and an empirical growth-rate derived by central differences; experimental SEs can be used as weights.
- Initial population matrix and bounds are saved / updated between bootstrap iterations to seed subsequent runs.

**End pseudo-code.**

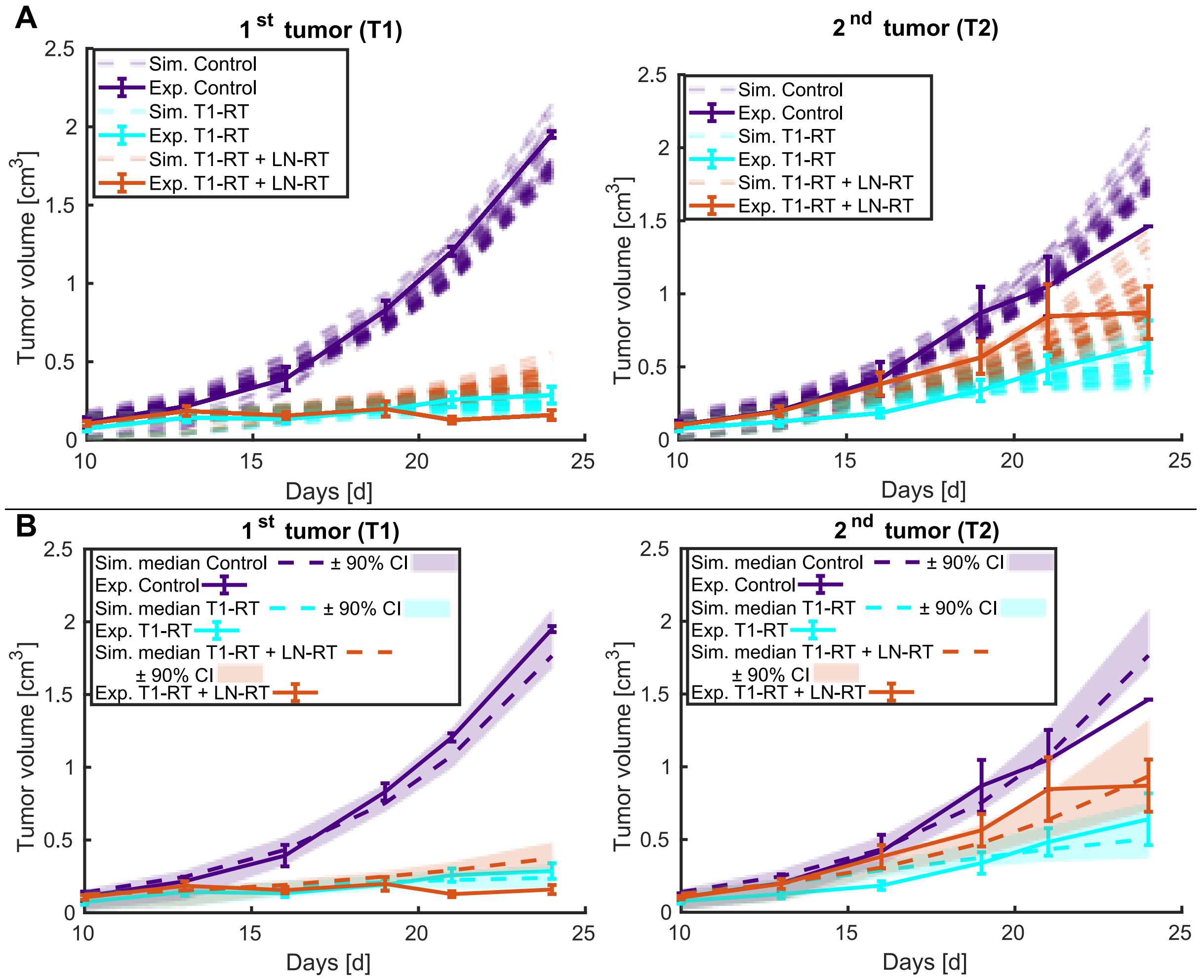


**Supplementary Figure 2: Model validation with experimental data. A) all individual simulated curves and B) the median and 90% of confidence interval from the 50 bootstrap resampling and model refit process.**

## 4 PBPK model parameters taken from literature

The rest parameters of the PBPK model are physiological and literature-derived and are presented in Supplementary Table 3. Supplementary Table 3 presents only the parameters that were not zero and their values were derived from [1–3].

**Supplementary Table 3: Physiological and literature-derived model parameters.**

| Category | Parameter | Value | Description |
|---|---|---|---|
| **Blood flows** | $Q_{blood}$ | 4.38 [cm³/min] | Blood flow rate |
| | $Q_{bone}$ | 0.17 [cm³/min] | Bone blood flow |
| | $Q_{heart}$ | 0.28 [cm³/min] | Heart blood flow |
| | $Q_{kidney}$ | 0.80 [cm³/min] | Kidney blood flow |
| | $Q_{liver}$ | 1.10 [cm³/min] | Liver blood flow |
| | $Q_{liver_{in}}$ | Q_liver+Q_gi-L_gi+Q_spleen-L_spleen | Effective liver inflow |
| | $Q_{muscle}$ | 0.80 [cm³/min] | Muscle blood flow |
| | $Q_{skin}$ | 1.21 [cm³/min] | Skin blood flow |
| | $Q_{spleen}$ | 0.05 [cm³/min] | Spleen blood flow |
| | $Q_{gi}$ | 0.90 [cm³/min] | GI tract blood flow |
| | $Q_{lnode}$ | 0.05 [cm³/min] | Lymph node blood flow |
| **Lymph flows** | $L_{blood}$ | 0 [cm³/min] | Blood lymph flow |
| | $L_{bone}$ | $6\times10^{-5}$ [cm³/min] | Bone lymph flow |

| | | | |
|---|---|---|---|
| | $L_{heart}$ | $1\times10^{-5}$ [cm³/min] | Heart lymph flow |
| | $L_{kidney}$ | $1.7\times10^{-4}$ [cm³/min] | Kidney lymph flow |
| | $L_{liver}$ | $2\times10^{-4}$ [cm³/min] | Liver lymph flow |
| | $L_{lung}$ | $1\times10^{-4}$ [cm³/min] | Lung lymph flow |
| | $L_{muscle}$ | $6\times10^{-4}$ [cm³/min] | Muscle lymph flow |
| | $L_{skin}$ | $1\times10^{-5}$ [cm³/min] | Skin lymph flow |
| | $L_{spleen}$ | $2\times10^{-6}$ [cm³/min] | Spleen lymph flow |
| | $L_{gi}$ | $7\times10^{-4}$ [cm³/min] | GI lymph flow |
| | $L_{lnode}$ | $1.8\times10^{-3}$ [cm³/min] | Lymph node lymph flow |
| **Vascular volumes** | $V_{v_{blood}}$ | 0.774 [cm³] | Blood vascular volume |
| | $V_{v_{bone}}$ | 0.080 [cm³] | Bone vascular volume |
| | $V_{v_{heart}}$ | 0.007 [cm³] | Heart vascular volume |
| | $V_{v_{kidney}}$ | 0.030 [cm³] | Kidney vascular volume |
| | $V_{v_{liver}}$ | 0.095 [cm³] | Liver vascular volume |
| | $V_{v_{lung}}$ | 0.019 [cm³] | Lung vascular volume |
| | $V_{v_{muscle}}$ | 0.150 [cm³] | Muscle vascular volume |
| | $V_{v_{skin}}$ | 0.200 [cm³] | Skin vascular volume |
| | $V_{v_{spleen}}$ | 0.010 [cm³] | Spleen vascular volume |
| | $V_{v_{gi}}$ | 0.100 [cm³] | GI vascular volume |

| | | | |
|---|---|---|---|
| | $V_{v_{lnode}}$ | 0.010 [cm³] | Lymph node vascular volume |
| **Interstitial volumes** | $V_{i_{blood}}$ | 0 [cm³] | Blood interstitial volume |
| | $V_{i_{bone}}$ | 0.280 [cm³] | Bone interstitial volume |
| | $V_{i_{heart}}$ | 0.019 [cm³] | Heart interstitial volume |
| | $V_{i_{kidney}}$ | 0.101 [cm³] | Kidney interstitial volume |
| | $V_{i_{liver}}$ | 0.190 [cm³] | Liver interstitial volume |
| | $V_{i_{lung}}$ | 0.057 [cm³] | Lung interstitial volume |
| | $V_{i_{muscle}}$ | 1.032 [cm³] | Muscle interstitial volume |
| | $V_{i_{skin}}$ | 0.999 [cm³] | Skin interstitial volume |
| | $V_{i_{spleen}}$ | 0.020 [cm³] | Spleen interstitial volume |
| | $V_{i_{gi}}$ | 0.600 [cm³] | GI interstitial volume |
| | $V_{i_{lnode}}$ | 0.020 [cm³] | Lymph node interstitial volume |
| **Radiotherapy time constant** | $\Delta\tau$ | 5 [min] | Time interval that radiotherapy term is activated |
| **Immune cell trafficking – APCs** | $J_{APC_{liver}}$ | 2.9×10⁻³ [1/min] | APC trafficking to liver |
| | $J_{APC_{lung}}$ | 2.9×10⁻³ [1/min] | APC trafficking to lung |
| | $J_{APC_{spleen}}$ | 2.9×10⁻³ [1/min] | APC trafficking to spleen |
| | $J_{APC_{lnode}}$ | 2.9×10⁻³ [1/min] | APC trafficking to lymph node |

| | | | |
|---|---|---|---|
| | $J_{APC_{tumor}}$ | 2.9×10$^{-3}$ [1/min] | APC trafficking to tumor |
| | $delta_{APC_{liver}}$ | 1 | Recirculation fraction of APCs from liver to lymph nodes |
| | $delta_{APC_{lung}}$ | 1 | Recirculation fraction of APCs from lung to lymph nodes |
| | $delta_{APC_{spleen}}$ | 1 | Recirculation fraction of APCs from spleen to lymph nodes |
| | $delta_{APC_{lnode}}$ | 1.8×10$^{-8}$ | Recirculation fraction of APCs from lymph nodes to blood entering the lungs |
| | $delta_{APC_{tumor}}$ | 0.1 | Recirculation fraction of APCs from tumor to lymph nodes |
| **Activation fraction of APCs after phagocytosis of the tumor** | $x_{DC}$ | 0.75 | DC to APC fraction |
| | $x_{M1}$ | 0.75 | M1 to APC fraction |
| **Immune cell trafficking – DCs** | $J_{DC_{liver}}$ | 2.9×10$^{-3}$ [1/min] | DC trafficking to liver |
| | $J_{DC_{lung}}$ | 2.9×10$^{-3}$ [1/min] | DC trafficking to lung |
| | $J_{DC_{spleen}}$ | 2.9×10$^{-3}$ [1/min] | DC trafficking to spleen |
| | $J_{DC_{tumor}}$ | 1.6617×10$^{-5}$ [1/d] | DC trafficking to tumor |
| | $delta_{DC_{bone}}$ | 1 | Recirculation fraction of DCs in bone |

| | | | |
|---|---|---|---|
| | $delta_{DC_{liver}}$ | 1 | Recirculation fraction of DCs from liver to lymph nodes |
| | $delta_{DC_{lung}}$ | 1 | Recirculation fraction of DCs from lung to lymph nodes |
| | $delta_{DC_{spleen}}$ | 1 | Recirculation fraction of DCs from spleen to lymph nodes |
| | $delta_{DC_{lnode}}$ | 1 | Recirculation fraction of DCs from lymph nodes to blood entering the lungs |
| | $delta_{DC_{tumor}}$ | 0.1 | Recirculation fraction of DCs from tumor to lymph nodes |
| | $phi_{DC_{bone}}$ | 6.2 [1/d] | DC proliferation rate in bone |
| **Immune cell trafficking – M1 macrophages** | $J_{M1_{liver}}$ | $2.9 \times 10^{-3}$ [1/min] | M1 trafficking to liver |
| | $J_{M1_{lung}}$ | $2.9 \times 10^{-3}$ [1/min] | M1 trafficking to lung |
| | $J_{M1_{spleen}}$ | $2.9 \times 10^{-3}$ [1/min] | M1 trafficking to spleen |
| | $J_{M1_{tumor}}$ | 1.01 [1/d] | M1 trafficking to tumor |
| | $delta_{M1_{bone}}$ | 1 | Recirculation fraction of M1 in bone |
| | $delta_{M1_{liver}}$ | 1 | Recirculation fraction of M1 from liver to lymph nodes |
| | $delta_{M1_{lung}}$ | 1 | Recirculation fraction of M1 from lung to lymph nodes |
| | $delta_{M1_{spleen}}$ | 1 | Recirculation fraction of M1 from spleen to lymph nodes |

| | | | |
|---|---|---|---|
| | $delta_{M1_{lnode}}$ | 1 | Recirculation fraction of M1 from lymph nodes to blood entering the lungs |
| | $delta_{M1_{tumor}}$ | 0.1 | Recirculation fraction of M1 from tumor to lymph nodes |
| | $phi_{M1_{bone}}$ | 6.2 [1/d] | M1 proliferation in bone |
| **Immune cell trafficking – M2 macrophages** | $J_{M2_{liver}}$ | $2.9\times10^{-3}$ [1/min] | M2 trafficking to liver |
| | $J_{M2_{lung}}$ | $2.9\times10^{-3}$ [1/min] | M2 trafficking to lung |
| | $J_{M2_{spleen}}$ | $2.9\times10^{-3}$ [1/min] | M2 trafficking to spleen |
| | $J_{M2_{tumor}}$ | $1.0578\times10^{-6}$ [1/d] | M2 trafficking to tumor |
| | $delta_{M2_{bone}}$ | 1 | Recirculation fraction of M2 in bone |
| | $delta_{M2_{liver}}$ | 1 | Recirculation fraction of M2 from liver to lymph nodes |
| | $delta_{M2_{lung}}$ | 1 | Recirculation fraction of M2 from lung to lymph nodes |
| | $delta_{M2_{spleen}}$ | 1 | Recirculation fraction of M2 from spleen to lymph nodes |
| | $delta_{M2_{lnode}}$ | 1 | Recirculation fraction of M2 from lymph nodes to blood entering the lungs |
| | $delta_{M2_{tumor}}$ | 0.1 | Recirculation fraction of M2 from tumor to lymph nodes |
| | $phi_{M2_{bone}}$ | 0.86 [1/d] | M2 proliferation in bone |

| | | | |
|---|---|---|---|
| **Immune cell trafficking – Tregs** | $J_{Treg_{liver}}$ | 2.9×10$^{-3}$ [1/min] | Treg trafficking to liver |
| | $J_{Treg_{lung}}$ | 2.9×10$^{-3}$ [1/min] | Treg trafficking to lung |
| | $J_{Treg_{spleen}}$ | 2.9×10$^{-3}$ [1/min] | Treg trafficking to spleen |
| | $J_{Treg_{tumor}}$ | 6024.8322 [1/d] | Treg trafficking to tumor |
| | $delta_{Treg_{bone}}$ | 1 | Recirculation fraction of Tregs in bone |
| | $delta_{Treg_{liver}}$ | 1 | Recirculation fraction of Tregs from liver to lymph nodes |
| | $delta_{Treg_{lung}}$ | 1 | Recirculation fraction of Tregs from lung to lymph nodes |
| | $delta_{Treg_{spleen}}$ | 1 | Recirculation fraction of Tregs from spleen to lymph nodes |
| | $delta_{Treg_{lnode}}$ | 1 | Recirculation fraction of Tregs from lymph nodes to blood entering the lungs |
| | $phi_{Treg_{blood}}$ | 1.736×10$^{-2}$ [cm$^3$/d] | Treg proliferation rate |
| **Immune cell trafficking – CD8$^+$ T cells (TE1)** | $J_{TE1_{liver}}$ | 2.9×10$^{-3}$ [1/min] | Effector CD8$^+$ T trafficking to liver |
| | $J_{TE1_{lung}}$ | 2.9×10$^{-3}$ [1/min] | Effector CD8$^+$ T trafficking to lung |
| | $J_{TE1_{spleen}}$ | 2.9×10$^{-3}$ [1/min] | Effector CD8$^+$ T trafficking to spleen |

| | | | |
|---|---|---|---|
| | $J_{TE1_{tumor}}$ | 14.671 [1/d] | Effector CD8$^+$ T trafficking to tumor |
| | $delta_{TE1_{liver}}$ | 1 | Recirculation fraction of TE from liver to lymph nodes |
| | $delta_{TE1_{lung}}$ | 1 | Recirculation fraction of TE from lung to lymph nodes |
| | $delta_{TE1_{spleen}}$ | 1 | Recirculation fraction of TE from spleen to lymph nodes |
| | $delta_{TE1_{lnode}}$ | 0.00119 [1] | Recirculation fraction of TE from lymph nodes to blood entering the lungs |
| **Tumor cell-induced M2** | $x_{M1_{to_{M2}}}$ | 7.51×10$^{-6}$ [cm$^3$/d] | Transition rate of M1 to M2 macrophages |
| **Suppression of effector cells by M2 and Tregs** | $k_{sup_{M2_{TE1}}}$ | 3.78 [cm$^3$/d] | Suppression of CD8+ effector T cells by M2 macrophages |
| | $k_{sup_{Treg_{TE1}}}$ | 4.35×10$^{-8}$ [cm$^3$/d] | Suppression of CD8+ effector T cells by Tregs |
| | $k_{sup_{M2_{APC}}}$ | 2.65 [cm$^3$/d] | Suppression of APCs by M2 macrophages |
| | $k_{sup_{Treg_{APC}}}$ | 1.86 [cm$^3$/d] | Suppression of APCs by Tregs |

## 5 Code availability

The COMSOL Multiphysics model is available online as a MATLAB script (https://doi.org/10.5281/zenodo.18624161)

## 6 References

[1] Nikmaneshi MR, Baish JW, Zhou H, Padera TP, Munn LL. Transport Barriers Influence the Activation of Anti-Tumor Immunity: A Systems Biology Analysis. Advanced Science 2023;10:2304076. https://doi.org/10.1002/ADVS.202304076.

[2] Zhu H, Melder RJ, Baxter LT, Jain RK. Physiologically based kinetic model of effector cell biodistribution in mammals: implications for adoptive immunotherapy. Cancer Res 1996;56:3771–81.

[3] Hadjigeorgiou AG, Munn LL, Stylianopoulos T, Jain RK. Physiologically based pharmacokinetic model for CAR-T cell delivery and efficacy in solid tumors. Proceedings of the National Academy of Sciences 2025;122:e2522634122. https://doi.org/10.1073/PNAS.2522634122.
